# FoxO-KLF15 pathway switches the flow of macronutrients under the control of insulin

**DOI:** 10.1016/j.isci.2021.103446

**Published:** 2021-11-15

**Authors:** Yoshinori Takeuchi, Naoya Yahagi, Yuichi Aita, Zahra Mehrazad-Saber, Man Hei Ho, Yiren Huyan, Yuki Murayama, Akito Shikama, Yukari Masuda, Yoshihiko Izumida, Takafumi Miyamoto, Takashi Matsuzaka, Yasushi Kawakami, Hitoshi Shimano

**Affiliations:** 1 Nutrigenomics Research Group, Faculty of Medicine, University of Tsukuba, 1-1-1 Tennodai, Tsukuba, Ibaraki 305-8575, Japan; 2 Department of Internal Medicine (Endocrinology and Metabolism), Faculty of Medicine, University of Tsukuba, Tsukuba, Ibaraki 305-8575, Japan

**Keywords:** Molecular biology, Molecular network, Diabetology

## Abstract

KLF15 is a transcription factor that plays an important role in the activation of gluconeogenesis from amino acids as well as the suppression of lipogenesis from glucose. Here we identified the transcription start site of liver-specific KLF15 transcript and showed that FoxO1/3 transcriptionally regulates *Klf15* gene expression by directly binding to the liver-specific *Klf15* promoter. To achieve this, we performed a precise *in vivo* promoter analysis combined with the genome-wide transcription-factor-screening method “TFEL scan”, using our original Transcription Factor Expression Library (TFEL), which covers nearly all the transcription factors in the mouse genome. Hepatic *Klf15* expression is significantly increased via FoxOs by attenuating insulin signaling. Furthermore, FoxOs elevate the expression levels of amino acid catabolic enzymes and suppress SREBP-1c via KLF15, resulting in accelerated amino acid breakdown and suppressed lipogenesis during fasting. Thus, the FoxO-KLF15 pathway contributes to switching the macronutrient flow in the liver under the control of insulin.

## Highlights

• The liver-specific transcript of KLF15 was identified
• *in vivo* Ad-luc and TFEL scan identified FoxOs as the upstream regulator of KLF15
• FoxOs act as a bridge between insulin signaling and KLF15
• FoxO-KLF15 pathway switches the macronutrient flow under the control of insulin

## Graphical abstract

**Figure:**
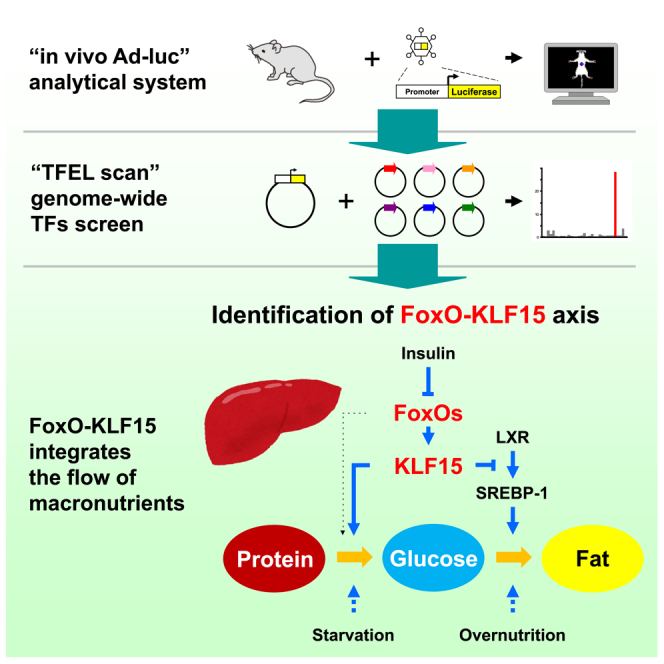

## Introduction

Three major essential macronutrients, protein, carbohydrates, and fat, are controlled by an integrated and well-balanced energy supply system *in vivo*. Although protein can be converted into carbohydrates and carbohydrates can be converted into fat, it is also well known that these macronutrients are not always interchangeable because the conversions are unidirectional and reverse conversions (from fat to carbohydrates and from carbohydrates to protein) cannot be made in animals. Therefore, the conversions among macronutrients are tightly regulated depending on the energy demand and nutritional environment.

Sterol regulatory element-binding protein-1c (SREBP-1c) is one of the transcription factors responsible for macronutrient balancing, and plays an important role in the lipogenesis, conversion of glucose into fat, in the liver (Shimano et al., 1997; Yahagi et al., 1999, 2002; Yokoyama et al., 1993). Fasting significantly reduces mRNA expression of SREBP-1c (gene name, *Srebf1c*) and reduces the amount of nuclear SREBP-1c protein in liver. Correspondingly, the mRNA of SREBP-1c target genes such as fatty acid synthase (FAS encoded by *Fasn*) is suppressed (Horton et al., 1998). It is a well-established mechanism that Liver X receptors (LXRs), members of the nuclear receptor family, transcribably regulate *Srebf1c* gene expression (Repa et al., 2000; Yoshikawa et al., 2001). In addition, it has been shown that insulin-dependent (Chen et al., 2004; Kim et al., 1998; Shimomura et al., 1999; Sun et al., 2016) and insulin-independent (Haas et al., 2012; Matsuzaka et al., 2004) mechanisms regulate SREBP-1c levels.

Recently, we have revealed that Krüppel-like factor 15 (KLF15) interacts with LXR to repress *Srebf1c* gene transcription as the essential mechanism of the nutritional regulation in the liver (Takeuchi et al., 2016). Hepatic KLF15 is rapidly induced during fasting, and also known to contribute to the regulation of hepatic gluconeogenesis, amino acid catabolism, endobiotic metabolism, and xenobiotic metabolism (Fan et al., 2018; Gray et al., 2007; Han et al., 2019; Jeyaraj et al., 2012b; Takashima et al., 2010; Teshigawara et al., 2005).

KLF15 is widely expressed in various tissues (Du et al., 2009; Gray et al., 2002; Haldar et al., 2012; Han et al., 2015; Jeyaraj et al., 2012a; Matoba et al., 2017; Uchida et al., 2000; Yamamoto et al., 2004), and in skeletal muscle and adipose tissue, the expression of KLF15 is regulated by glucocorticoid receptor (GR), a member of the nuclear receptor family (Asada et al., 2011; Morrison-Nozik et al., 2015; Shimizu et al., 2011). However, the regulatory mechanism in the liver remains largely unknown.

Here we show that FoxO1 and FoxO3a (FoxOs), the forkhead box O-class (FoxO) subfamily of the forkhead transcription factors, regulate the transcription of *Klf15-1a*, a liver-specific *Klf15* transcript. To achieve this, we performed a precise *in vivo* promoter analysis using “*in vivo* Ad-luc” analytical system (Takeuchi et al., 2016), combined with the genome-wide screening method “TFEL scan”, using our original cDNA library named Transcription Factor Expression Library (TFEL), which is composed of nearly all the transcription factors in the mouse genome (Yahagi and Takeuchi, 2021).

FoxOs transcriptional activities are known to be regulated through the phosphorylation of their proteins by Akt, a kinase in the insulin signaling pathway (Accili and Arden, 2004; Brunet et al., 1999; Haeusler et al., 2018; Matsuzaki et al., 2003; Puigserver et al., 2003; Rena et al., 1999). Thus, it is well established that the insulin-FoxO pathway controls glucose and lipid metabolism (Deng et al., 2012; Haeusler et al., 2014; Matsumoto et al., 2006; Nakae et al., 2001; Zhang et al., 2006, 2012).

This report demonstrates that the hepatic FoxO-KLF15 axis regulates both gluconeogenesis from amino acids and lipogenesis from glucose, integrating the unidirectional conversions of macronutrients under the control of insulin.

## Results

### Identification of TSS of liver-specific *Klf15* transcript

Although it is known that the *Klf15* gene is highly expressed in the liver and that alternatively spliced variants have been reported, it is not clear how *Klf15* transcription initiates (Du et al., 2009; Teshigawara et al., 2005). Therefore, we performed RNA-seq analysis using liver mRNA. As shown in Figure S1A, few transcripts from conventional exon 1 were detectable and a vigorous peak within the intron1 region between exon1 and exon 2 was observed. This peak is considered to be the transcript corresponding to human *KLF15* exon 1a that was previously reported as a component of *KLF15-1a* liver-specific variant, while the conventional *Klf15* exon 1 was defined as exon 1c (Du et al., 2009). To confirm the RNA-seq data, we performed Q-RT PCR with primer sets shown in Figure S1A to compare the amounts of exon 1c and 1a in several tissues where the role of KLF15 is well known. As shown in Figures S1B and S1C, *Klf15-1a* was expressed only in the liver, whereas *Klf15-1c* was detected in all the investigated tissues. Three major peaks of RNA polymerase II (Pol II), modified histones H3K4Me3, H3K9Ac, and H3K27Ac binding were observed around the exon 1a in the public ChIP-seq data from Bing Ren's laboratory at the Ludwig Institute for Cancer Research (LICR) obtained on the genome browser (https://genome.ucsc.edu/). Among them, one is seen at the position of exon 1a and another is located at the position of exon 1c (Figure S1D). Furthermore, to evaluate a detailed transcriptional start site (TSS) for *Klf15-1a*, we performed RNA-seq analysis using unspliced pre-mRNA isolated from liver nuclei. Although there might be several potential TSS between exons 1a and 1c, no other transcripts were detected by Q-RT PCR using the indicated various primer sets (Figures S1E and S1F). In addition, the transcript containing both exon 1c and exon 1a was not detected. From these results, we concluded that the *Klf15* gene has a structure shown in Figure S1G.

Because *Klf15* expression is known to increase during fasting and decrease in the postprandial state, we examined the *Klf15-1a* expression in mouse liver in fasted and re-fed states. As shown in Figure S1H, both *Klf15-1a* and *Klf15-1c* expressions similarly increased in the fasted state and suppressed in the re-fed state, and both in fasted and re-fed states, *Klf15-1a* transcript accounted for the main proportion. These results demonstrate that *Klf15-1a* is the major variant that plays an important role in the liver.

### Finding two important cis-elements to control expression of liver-specific *Klf15* transcript during fasting

**Figure 1:**
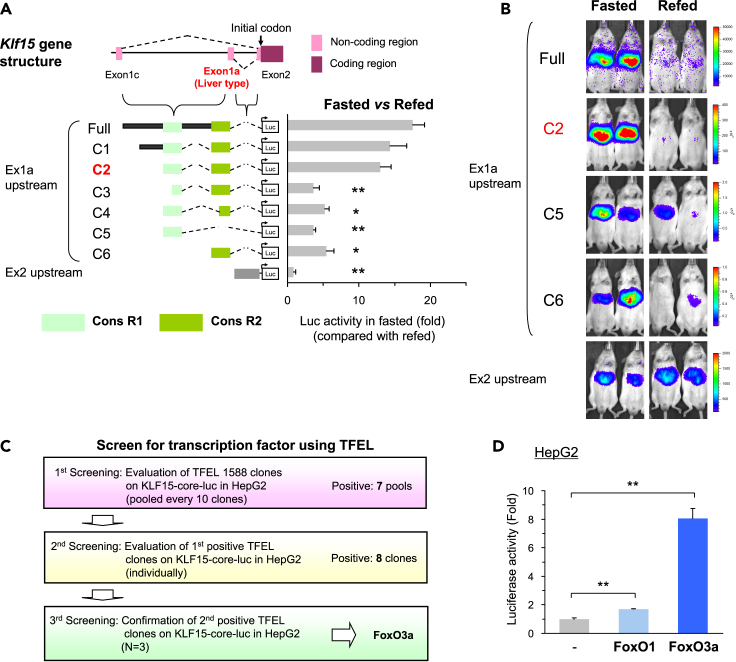
Identification of liver-specific *Klf15* transcript and its regulatory genomic regions by fasting (A and B) *in vivo* Ad-luc promoter analyses to determine genomic region for *Klf15* gene regulation in fasted and re-fed states. The *Klf15* gene structure is shown in the upper diagram. Hepatic luciferase activities (A) and representative images (B) of mice injected with various Ad-*Klf15*-Luc are shown (n = 5–14). “Full” of Ex1a upstream Ad-luc construct contains the entire region between exon 1a and 1c. Construct #2 (C2) was named as *Klf15*-core-luc. The fasting promoter activity in the liver is expressed relative to activity in the refed state, to adjust for mouse-to-mouse differences in promoter expression in Ad-treated mice. (C) Processes of screening to identify transcription factors that bind to *Klf15*-core regions using TFEL (Transcription Factor Expression Library). (D) Identification of FoxO1 and FoxO3a transcription factors as regulators of *Klf15*-core-luc (n = 3). TFEL clones expressing FoxO1 and FoxO3a were co-transfected with *Klf15*-core-luc in HepG2 cells. Datasets were assessed by ANOVA. The differences were considered to be significant if *P* < 0.05. (∗*P* < 0.05 and ∗∗*P* < 0.01).

We have previously developed an intra-organ assay system named “*in vivo* Ad-luc” analytical system to elucidate the transcriptional regulation mechanism *in vivo* using a recombinant adenovirus containing a genomic region of interest fused to firefly luciferase reporter gene (Murayama et al., 2019; Nishi-Tatsumi et al., 2017; Takeuchi et al., 2007, 2010, 2016). Based on the gene structure described above, we first generated two kinds of Ad-luc constructs using 2.7k upstream of exon 1a (Ex1a) or 0.5kb upstream of exon 2 (Ex2) as shown in Figure 1A and evaluated the transcriptional activity in the fasted and re-fed states. Ad-luc constructs were transduced into mouse liver, and transcriptional activity was assessed by measuring luciferase activity with an IVIS imaging system. As shown in Figures 1A and 1B, Ex1a upstream 2.7k construct (labeled “Full”) had a substantial increase of the luciferase activity during fasting similar to endogenous *Klf15-1a* gene expression.

Next we proceeded to a deletion study; to identify the regulatory cis element on the *Klf15-1a* promoter activated by fasting using *in vivo* Ad-luc analytical system, we made various Ad-luc constructs containing various length of *Klf15-1a* promoter region (Full and C1-5) as described in Figures 1A and 1B. From these results, two regions conserved among mammalian species, named Cons R1 and Cons R2, were identified as the important cis-elements for fasting responses. Intriguingly, each of the two elements alone was not sufficient, but as long as the two are both present, the order does not matter, because the responses were not affected if the elements were placed in the opposite order as shown in Figures S1I and S1J. Based on the finding that the Ad-*Klf15*-luc (C2) contained the necessary and sufficient cis-elements, we further proceeded to identification of trans-factors using this construct (designated as *Klf15*-core-luc).

### FoxO binding sites are important for *Klf15-1a* promoter regulation

**Figure 2:**
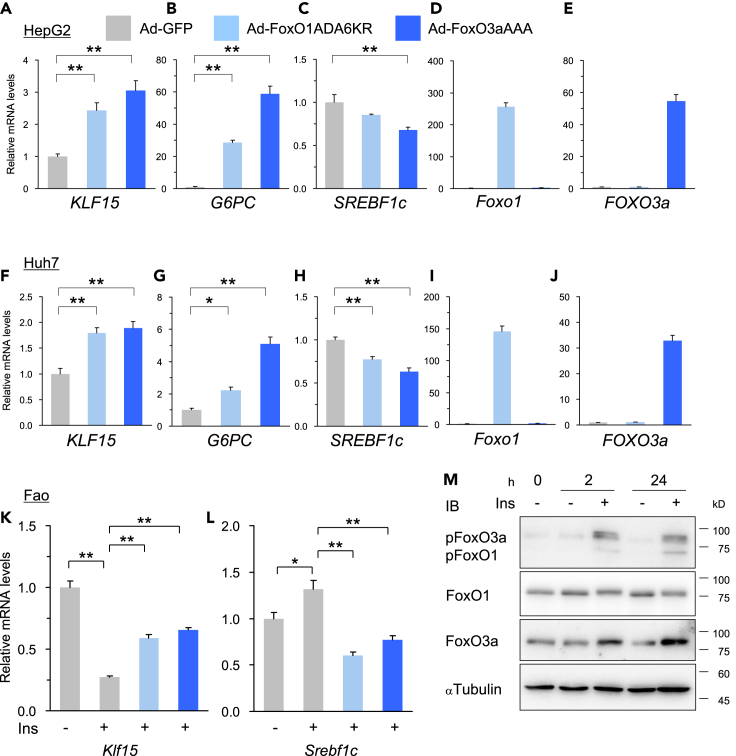
FoxO1 and FoxO3a are important for *Klf15* expression in hepatocytes (A–J) Q-RT PCR analysis of the indicated genes in human hepatoma cell lines. Constitutive active FoxO1 and FoxO3a were over-expressed in HepG2 (A–E) and Huh7 (H–J) cells using adenoviruses (Ad-FoxO1ADA6KR and FoxO3aAAA, respectively) for 24-hr (n = 4–5). (K–M) Rescue experiments of FoxO1 and FoxO3a on *Klf15* suppression by insulin in Fao rat hepatoma cell lines. The cells were starved in a serum-free medium containing 0.01 nM insulin and 10 nM dexamethasone for 2-hr, and then the medium was changed to be treated with 100 nM insulin for 24-hr. Q-RT PCR analysis of *Klf15* (K) and *Srebf1c* (L) genes in insulin treated Fao cells. FoxO1ADA6KR and FoxO3aAAA were over-expressed in Fao cells using adenoviruses for 24-hr before insulin treatment (n = 5). Immunoblot analysis of phospho-FoxO1 and Phospho-FoxO3a proteins in insulin treated Fao cells for the indicated time (M). Datasets were assessed by ANOVA. The differences were considered to be significant if *P* < 0.05. (∗*P* < 0.05 and ∗∗*P* < 0.01).

To identify the trans-factors corresponding to the above identified cis-elements, we screened 1,588 genome-wide transcription factor genes included in our original cDNA library named Transcription Factor Expression Library (TFEL) as described previously (Piao et al., 2018; Takeuchi et al., 2016; Yahagi and Takeuchi, 2021). After three rounds of screening in HepG2 cells as shown in Figures 1C and S2A–S2C, FoxO3a transcription factor was identified as a candidate with the ability to up-regulate the luciferase activity of *Klf15*-core-luc. FoxO3a (also known as FKHRL1) belongs to the forkhead box O-class (FoxO) subfamily of the forkhead transcription factors, and known to be involved in many physiological and pathological processes including glucose and lipid metabolisms in the liver (Haeusler et al., 2014; Nakae et al., 2001; Zhang et al., 2006, 2012). Because another FoxO subfamily member FoxO1 is known to have functions similar to FoxO3a, we examined the ability to increase the luciferase activity of *Klf15*-core-luc. As shown in Figure 1D, FoxO1 enhances the luciferase activity similar to FoxO3a. Furthermore, to investigate whether FoxO1 and FoxO3a directly affect *Klf15* expression, adenoviruses that express constitutive active mouse FoxO1 and human FoxO3a (FoxO1ADA6KR and FoxO3aAAA, respectively) were transduced into HepG2 and Huh7 hepatoma cells, leading to a result that FoxO1 and FoxO3a also upregulated intrinsic *KLF15* expression similar to *G6PC* known as a FoxOs target in hepatocytes as shown in Figures 2A–2J. In contrast, *Srebf1c*, the critical regulator of lipogenesis in the liver, was significantly repressed and these results reinforce our previous finding that *Srebf1c* is a direct target of KLF15, suppressing lipogenesis during fasting (Takeuchi et al., 2016). To confirm the effect of insulin on FoxO-KLF15 pathway, Fao hepatoma cells, which have detectable insulin action similar to that of liver tissue (Kawata et al., 2018; Sano et al., 2016), were treated with insulin. Under the condition that intrinsic FoxO1 and FoxO3a were phosphorylated sustainably, it was demonstrated that insulin repressed *Klf15* expression as shown in Figures 2K–2M and S2D. In addition, the repressive effect was rescued by over-expression of FoxO1ADA6KR and FoxO3aAAA, which are not excluded from the nucleus by avoiding insulin-induced phosphorylation (Kitamura et al., 2005). In contrast, *Srebf1c* expression significantly increased by insulin and the effect was canceled by FoxO1ADA6KR and FoxO3aAAA overexpression.

### Role of FoxOs in *Klf15-1a* regulation in liver

**Figure 3:**
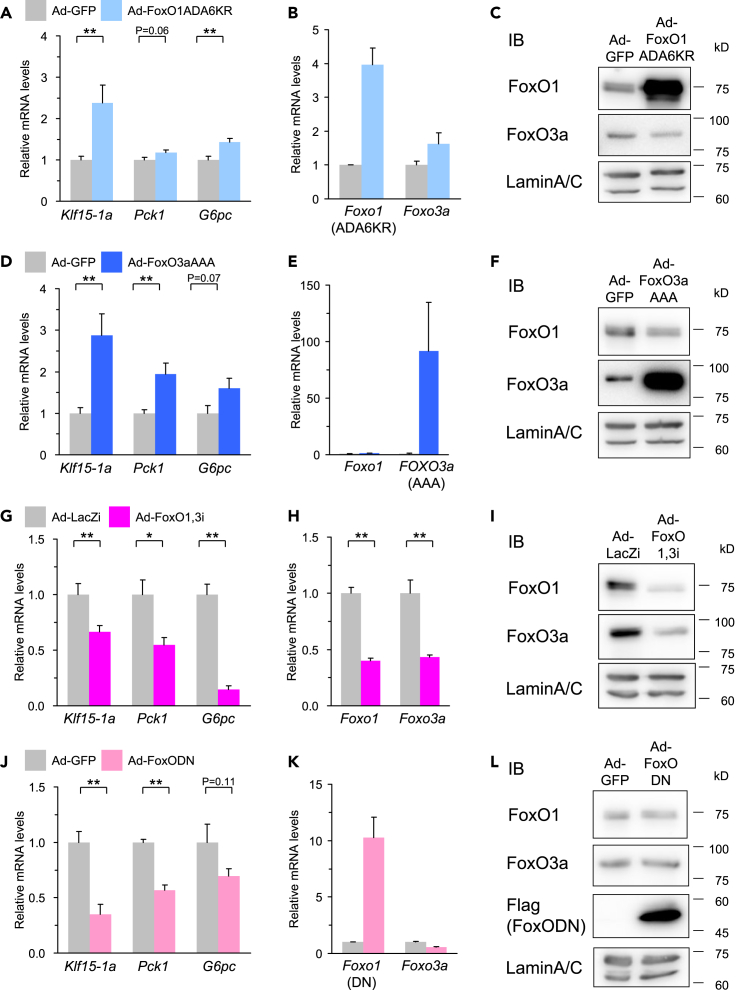
Role of FoxO1 and FoxO3a on *Klf15-1a* regulation in liver Q-RT PCR analysis of liver RNA samples and immunoblots using liver nuclear extracts in fasted mice. (A–F) Elevations of FoxO1 and FoxO3a. Constitutive active FoxO1 and FoxO3a (FoxO1ADA6KR (A–C) and FoxO3aAAA (D–F)) were over-expressed in liver using an adenovirus (Ad-FoxO1ADA6KR and FoxO3aAAA, respectively) (n = 6). (G–I) Knockdowns of FoxO1 and FoxO3a. Knockdowns of hepatic FoxO1 and FoxO3a were performed using adenovirus-mediated RNAi (Ad-FoxO1,3i) (n = 6). (J–L) Reductions of endogenous FoxO1 and FoxO3a transcription activities. Flag-tagged dominant-negative (DN) FoxO1 protein was over-expressed in liver using an adenovirus (Ad-FoxODN) (n = 6). Data in (A), (B), (D), (E), (G), (H), (J), and (K) are Q-RT PCR analysis of the indicated genes. Pck1 and G6pc are target genes of FoxO1 and FoxO3a. Data in (C), (F), (I), and (L) are immunoblots with the indicated antibodies (Samples were pooled from 3 - 4 mice). 2-days (A–F), 3-days (G–I) and 5-days (J–L) after the transduction of each adenovirus, mice were starved for 24-hr from the light phase. Data were assessed using the unpaired two-tailed Student's t-test. The differences were considered to be significant if *P* < 0.05. (∗*P* < 0.05 and ∗∗*P* < 0.01).

To clarify the role of FoxO proteins in the regulation of *Klf15-1a* gene expression in liver, we examined the influences of the gain-of-function and the loss-of-function of FoxO1 and FoxO3a using adenoviruses. As expected, overexpression of FoxO1ADA6KR and FoxO3aAAA in fasted mouse livers led to increases in the expressions of *Pck1* and *G6pc*, genes involved in gluconeogenesis and known as FoxO targets, and *Klf15-1a* simultaneously (Figures 3A–3F). Conversely, when we examined the contribution of FoxO1 and FoxO3a in the fasting state by knocking down using adenovirus expressing small hairpin RNAs (shRNAs) for *Foxo1* and *Foxo3a* (Ad-FoxO1,3i), *Pck1*, *G6pc,* and *Klf15-1a* were markedly suppressed despite fasting conditions (Figures 3G–3I). The adenovirus-mediated overexpression of dominant-negative FoxO1 (Ad-FoxODN) exerted essentially the same effects on expression levels of these genes to exclude the possibility of artificial effects (Figures 3J–3L). It has been reported that KLF15 plays a critical role in nitrogen homeostasis and amino acid metabolism in the liver (Gray et al., 2007; Jeyaraj et al., 2012b; Takashima et al., 2010). Therefore, we investigated the genes of various enzymes involved in amino acid metabolism, *Alt1*, *Prodh*, *Hpd,* and *Otc*, known as target genes regulated by KLF15, in the livers of Ad-FoxO1,3i (knockdown) and Ad-FoxODN (dominant-negative) models. As shown in Figures S3A and S3B, these genes were markedly suppressed in both loss-of-function models.

### Insulin-FoxO pathway is involved in the regulation of *Srebf1c* and amino acid metabolism genes via KLF15

**Figure 4:**
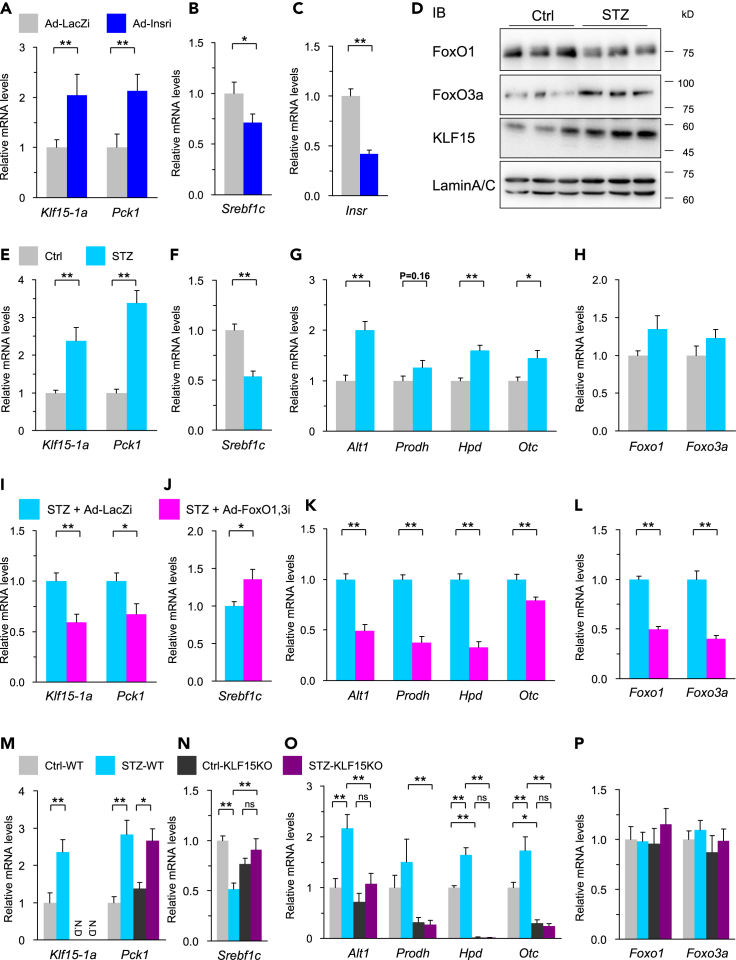
Insulin-FoxO pathway is involved in hepatic *Srebf1c* gene expression via KLF15 Q-RT PCR analysis of liver RNA samples and immunoblots using liver nuclear extracts in re-fed mice. (A–C) Q-RT PCR analysis of hepatic insulin receptor knockdown using adenovirus-mediated RNAi (Ad-Insri) (n = 8) Four days after the adenovirus transduction, mice were starved for 24-hr from the early dark phase, and then they were re-fed for 16-hr after the starvation. (D) Immunoblot analysis of FoxO1, FoxO3a and KLF15 using nuclear extracts from liver of insulin depleted mice by streptozotocin (STZ) administration (n = 3). (E–H) Q-RT PCR analysis of insulin-depleted diabetic mice by STZ (n = 8). (I–L) Q-RT PCR analysis of hepatic FoxO1 and FoxO3a knockdown samples using Ad-FoxO1,3i in insulin-depleted diabetic mice by STZ (n = 9–10). (M–P) Q-RT PCR analysis of liver samples from insulin-depleted diabetic KLF15KO mice by STZ (n = 8–11). The experimental procedure for STZ treated mice (d-p) is described in Figure S4A. *Alt1*, *Prodh*, *Hpd*, *Otc,* and *Srebf1c* are known as the genes regulated by KLF15 in liver. Differences between two groups were assessed using the unpaired two-tailed Student's t-test. Datasets involving more than two groups were assessed by ANOVA. The differences were considered to be significant if *P* < 0.05. (∗*P* < 0.05 and ∗∗*P* < 0.01).

To examine the effect of insulin on *Klf15-1a* expression via FoxOs in the liver *in vivo*, we analyzed hepatic insulin receptor knockdown model using the adenovirus expressing shRNA for *Insr* adenovirally (Ad-Insri). As shown in Figures 4A–4C, *Klf15-1a* and *Pck1* genes were increased by Ad-Insri. On the contrary, *Srebf1c* expression was significantly decreased. Next, we assessed the insulin-deficient mice treated with streptozotocin (STZ) according to the experimental procedure in Figure S4A. As shown in Figures 4D–4H, insulin depletion increased nuclear levels of FoxO3a, not FoxO1, resulting in *Klf15-1a* elevation and thereby the activation of KLF15-target amino acid metabolism genes such as *Alt1*, *Prodh*, *Hpd,* and *Otc*. STZ treatment markedly diminished plasma insulin levels and at the same time increased blood glucose levels, whereas Ad-Insri administration did not change blood glucose levels. Nevertheless, STZ and Ad-Insri caused essentially the same effects on expression levels of *Srebf1c*, suggesting that insulin signaling regulates *Klf15-1a* gene expression in the liver (Figures S4B–S4E). To evaluate whether the insulin effect on *Klf15-1a* was mediated by FoxOs, we compared the gene expressions in the livers of STZ mice with or without FoxO1 and FoxO3a knockdown. As shown in Figures 4I–4L, S4F, and S4G, hepatic FoxO1 and FoxO3a knockdown lowered the expressions of *Klf15-1a* and its target genes *Alt1*, *Prodh*, *Hpd,* and *Otc,* and also elevated *Srebf1c* expression, which increased triglycerides in the liver. Furthermore, to investigate whether the effects of insulin depletion were mediated by KLF15, we analyzed the gene expressions in the livers of KLF15KO mice treated with STZ. As shown in Figures 4M–4P, S4H, and S4I, the effects of insulin deficiency on the expression levels of *Srebf1c* and other KLF15-target amino acid metabolism genes including *Alt1*, *Prodh*, *Hpd,* and *Otc* were completely canceled in KLF15KO, demonstrating that the insulin regulation of *Srebf1c* expression is mediated via the FoxO-KLF15 pathway. These results indicate that the insulin-FoxO-KLF15 axis plays an integrative role in lipid and amino acid metabolism in the liver.

### FoxO protein directly binds to *Klf15-1a* promoter region

**Figure 5:**
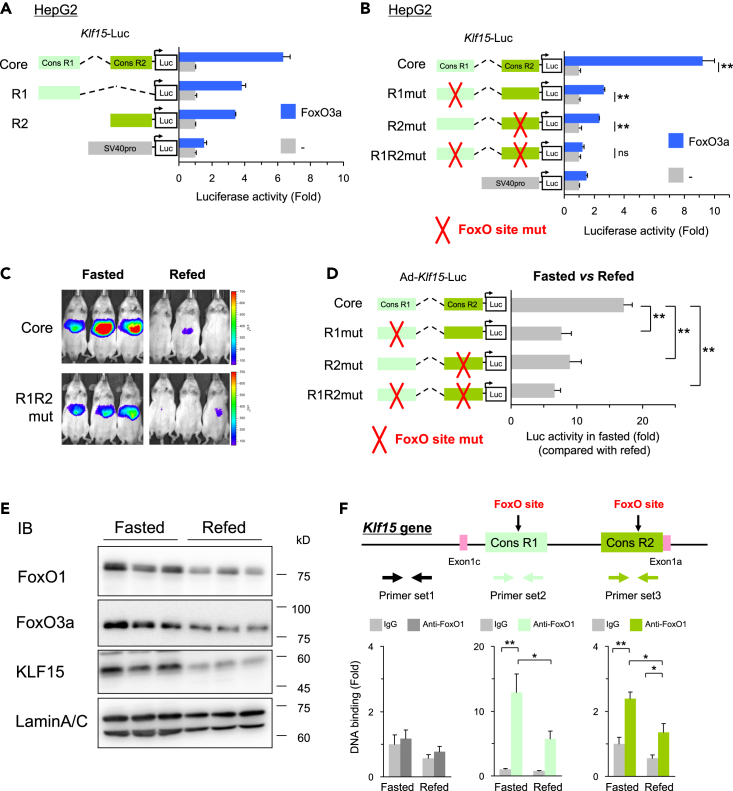
FoxO protein directly binds to *Klf15-1a* promoter regions. (A and B) Promoter analysis of FoxO3a on separated *Klf15*-core-luc (A) and FoxO binding site mutated *Klf15*-core-luc (B) (n = 3). FoxO3a expression plasmid was co-transfected with the indicated *Klf15*-core-luc plasmids in HepG2 cells. ∗∗represents *P* < 0.01, unpaired two-tailed Student's t-test. (C and D) *In vivo* Ad-luc promoter analyses using FoxO binding site mutated Ad-*Klf15*-luc. Images (C) and hepatic luciferase activities (D) of mice injected with Ad-*Klf15*-Luc are shown (n = 5–6). The fasting promoter activity in the liver is expressed relative to activity in the refed state, to adjust for mouse-to-mouse differences in promoter expression in Ad-treated mice. ∗∗represents *P* < 0.01, ANOVA. (E) Immunoblot analysis of FoxO1, FoxO3a, and KLF15 using liver nuclear extracts from fasted and re-fed mice (n = 3). (F) Elucidations of FoxO protein binding to *Klf15* promoter in liver. ChIP assay were performed with anti-FoxO1 antibody and normal IgG as a control using liver samples of fasted and re-fed mice (n = 8). Primer set2 and set3 were used for detection of FoxO binding sites on the *Klf15* promoter. Primer set1 was designed as a negative control. ∗ and ∗∗ represents *P* < 0.05 and *P* < 0.01 by ANOVA, respectively.

To determine the FoxOs binding sites in the *Klf15*-core promoter region, we performed the promoter analysis using various *Klf15*-luc constructs in HepG2. As shown in Figures S5A–S5D, JASPAR (http://jaspar.genereg.net/) predicted one FoxOs binding site in each of Cons R1 and R2 elements. When we mutated these predicted binding sites, the activation by FoxO3a was completely abolished as shown in Figure 5B. In addition, the result showed that both two FoxO-binding sites are needed, suggesting an interaction between the two sites. These results were further supported by an *in vivo* mutation analysis, which demonstrated that the mutations at the FoxO- binding sites markedly decreased the fasting response of the *Klf15*-core promoter activity as shown in Figures 5C and 5D.

Next, we examined nuclear FoxO1 and FoxO3a protein levels in the liver in the fasted and re-fed states. Transcriptional activities of FoxO1 and FoxO3a are known to be regulated by insulin-Akt pathway mediated phosphorylation and translocating from the nucleus to the cytosol. As shown in Figure 5E, the protein expression levels of nuclear FoxO1 and FoxO3a were higher in the fasted state compared with the re-fed state. Concomitantly, the abundance of KLF15 protein was also increased during fasting, consistent with previous reports (Takeuchi et al., 2016; Teshigawara et al., 2005). Furthermore, chromatin immunoprecipitation (ChIP) assays demonstrated that FoxO1 binding occupancy to the *Klf15-1a* promoter region was significantly increased in the fasting state compared with the re-fed state as shown in Figure 5F. It is well known that the non-coding genomic region conserved among species plays an important role for gene expression (Pennacchio and Rubin, 2001; Siepel et al., 2005), and the DNA sequences of the two FoxOs binding sites are highly conserved among mammalians as shown in Figures S5E and S5F. From these results, it is suggested that FoxO protein binds to the *Klf15-1a* promoter region and regulates the transcription of *Klf15-1a* in the liver during fasting when blood insulin levels decrease.

### Hepatic FoxOs are involved in amino acid metabolism via KLF15 pathway

**Figure 6:**
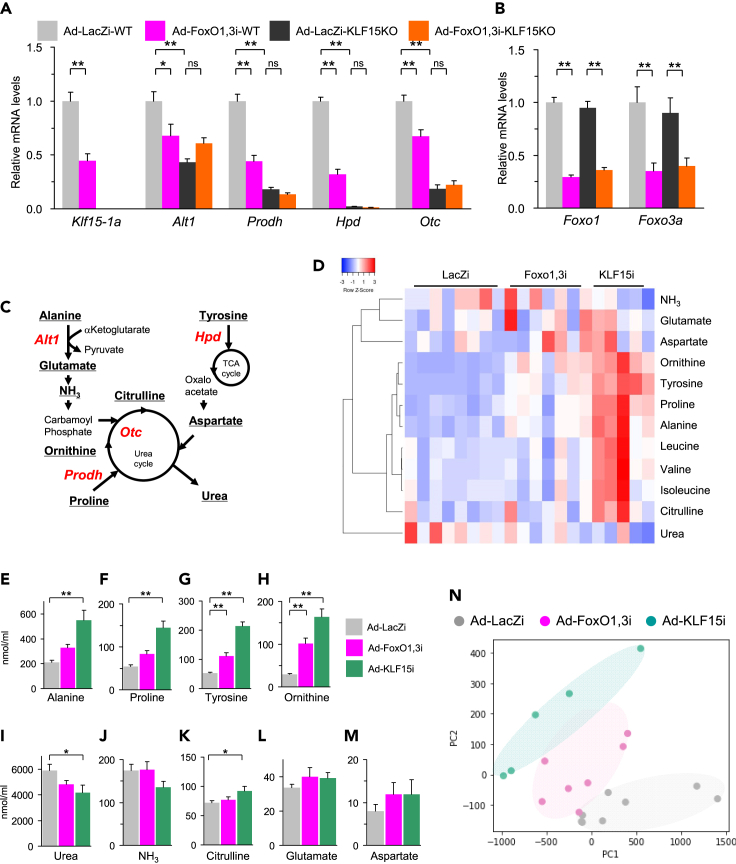
Hepatic FoxO1 and FoxO3a are involved in amino acid metabolism via KLF15 pathway (A and B) Q-RT PCR analysis of liver RNA samples from hepatic FoxO1 and FoxO3a knockdown KLF15KO mice using Ad-FoxO1,3i in the fasted state (n = 6–7). Four days after the adenovirus transduction, mice were starved for 24-hr from the light phase. (C) Schema of amino acid metabolic genes regulated by KLF15 in the liver. These genes, shown in red, are known to be regulated by KLF15 in the liver. (D–N) Heatmap of plasma amino acid composition (D) and each concentration related to KLF15 target genes (E–M) in hepatic FoxO1 + FoxO3a or KLF15 knockdown mice using adenoviruses after fasting (n = 5–8). Four days after the transduction of each adenovirus, mice were starved for 24-hr from the light phase. PCA (N) was calculated using all measured amino acid data. Datasets were assessed by ANOVA. The differences were considered to be significant if *P* < 0.05. (∗*P* < 0.05 and ∗∗*P* < 0.01).

**Figure 7:**
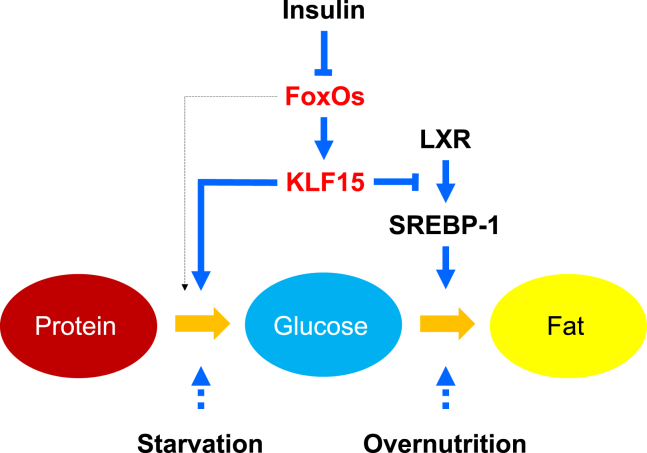
FoxO-KLF15 axis integrates glucose, lipid, and amino acid metabolism in liver Schematic presentation of the molecular mechanism by which the FoxO-KLF15 axis integrates glucose, lipid and amino acid metabolism in the liver.

FoxO1 and FoxO3a are well known to be involved in gluconeogenesis and lipid metabolism in the liver (Deng et al., 2012; Haeusler et al., 2014; Matsumoto et al., 2006; Nakae et al., 2001; Zhang et al., 2006, 2012). However, there are few data suggesting a link between FoxOs and amino acid metabolism. Therefore, to clarify the relationship between FoxO-KLF15 pathway and amino acid metabolism, we evaluated the effects of FoxOs knockdown using Ad-FoxO1,3i on the expression levels of genes involved in amino acid metabolism, and also compared the knockdown effects between wild-type and KLF15KO mice. As shown in Figures 6A and 6B, the suppressive effects of FoxOs knockdown on the amino acid metabolism-related KLF15 target genes (*Alt1*, *Prodh*, *Hpd* and *Otc*) were eliminated completely. In addition, in the experiments using primary hepatocytes from KLF15KO mice, the effects of over-expression of FoxO3aAAA on these target genes as well as on *Srebf1c* were also shown to be completely disrupted in KLF15KO hepatocytes (Figures S6A and S6B), demonstrating that the effects of FoxO are fully mediated through KLF15 as far as *Alt1, Prodh, Hpd, Otc, and Srebf1c* are concerned. The enzymes encoded by *Alt1*, *Prodh*, *Hpd,* and *Otc* genes play important roles in the amino acid degradation processes shown in Figure 6C; alanine is converted to pyruvate by alanine transaminase1 (ALT1 encoded by *Alt1*) via oxaloacetate, proline is converted to glutamate by proline dehydrogenase 1 (PRODH encoded by *Prodh*), tyrosine enters the TCA circuit via fumaric acid by 4-hydroxyphenylpyruvate dioxygenase (HPD encoded by *Hpd*), which provides carbon for gluconeogenesis, and the ornithine carbamoyltransferase (OTC encoded by *Otc*) gene produces citrulline from ornithine and carbamoyl phosphate in the urea circuit. To further evaluate the specific contribution of KLF15-mediated amino acid metabolic pathway in the liver, liver-specific KLF15 knockdown mice were prepared using adenovirus expressing the shRNA for *Klf15* (Ad-KLF15i) described previously (Takeuchi et al., 2016), instead of KLF15KO mice lacking the KLF15 gene throughout the body. Consequently, as shown in Figures 6D–6N and S6C–S6F, the plasma concentration of amino acids alanine, proline, tyrosine, and ornithine were elevated by FoxOs and KLF15 knockdown, corresponding to the lower expression level of each enzyme gene, *Alt1*, *Prodh*, *Hpd,* and *Otc*. These results demonstrate that the FoxO-KLF15 axis integrates glucose, lipid and amino acid metabolism in the liver (Figure 7).

## Discussion

This study shows that *Klf15* gene expression in the liver is regulated through the insulin-FoxO pathway and that the metabolic flow between the three macronutrients, protein, carbohydrate, and fat is integrally regulated by this pathway (Figure 7).

KLF15 is widely expressed in various tissues (Uchida et al., 2000) and the splicing variant *KLF15-1a* with the liver-specific exon 1a has been previously reported (Du et al., 2009). In the present study, we demonstrated that *Klf15-1a* is the major variant that plays an important role in the liver. Moreover, the mRNA expression of *Klf15-1a* increased during fasting, consistent with previous reports (Takeuchi et al., 2016; Teshigawara et al., 2005) (Figures 1A and 1B). Using our unique intra-organ promoter analysis technique “*in vivo* Ad-luc” analytical system (Murayama et al., 2019; Nishi-Tatsumi et al., 2017; Takeuchi et al., 2010, 2016), we identified two important genomic regions within an unexplored area for the fasting response of the *Klf15-1a* gene in liver (Figures 1A, 1B, S1I, and S1J); in the previous studies of *Klf15* gene promoter analysis (Asada et al., 2011; Shao et al., 2018; Shimizu et al., 2011), they analyzed only 1 k upstream and 1.3 k downstream regions of the exon 1c TSS, which did not cover the sufficient genomic regions required for the fasting response. Moreover, we clarified by a genome-wide screening method “TFEL scan” using our original cDNA library named Transcription Factor Expression Library (TFEL) covering nearly all the transcription factors in the mouse genome (Piao et al., 2018; Takeuchi et al., 2016; Yahagi and Takeuchi, 2021) that the genomic regions are activated by FoxOs (FoxO1 and FoxO3a) both in cultured hepatocytes (Figures 2A and 2F) and in mouse liver tissue (Figures 3A, 3D, 3G, and 3J). Furthermore, it was shown that the FoxO-binding sequences are important for transcriptional activation during fasting (Figures 5A–5D) and FoxOs binding to the two sites increases in correlation with its increase in nuclear protein abundance during fasting (Figures 5C–5F). Thus, we clarified that KLF15 is a FoxO target in the liver.

Although the fact that FoxOs control the hepatic expression of KLF15 is revealed for the first time in the present study, there are several similar cases known for other KLF family members; for example, it is reported that KLF4 is a FoxO target in B cell (Yusuf et al., 2008), and so is KLF2 in T cell (Fabre et al., 2008) and KLF6 in lung adenocarcinoma (Sangodkar et al., 2012).

It is reported in previous studies that insulin suppresses *Klf15* gene expression in hepatocytes and liver tissue (Kalvisa et al., 2018; Takeuchi et al., 2016; Teshigawara et al., 2005). It is also reported that the livers of liver-specific insulin receptor knockout (LIRKO) mice show elevated expression of KLF15 (Haas et al., 2012), consistent with our results (Figures 2K, 4A, and 4E). Thus, we succeeded in identifying FoxOs as the bridge between the insulin signaling pathway and KLF15.

It has been well established that the FoxO activity is regulated by its phosphorylation via Akt, a kinase in the insulin signaling pathway, which results in the sequestration of these transcription factors out of the nucleus and the subsequent degradation of the protein by the ubiquitin-proteasome system (Brunet et al., 1999; Haeusler et al., 2018; Matsuzaki et al., 2003; Puigserver et al., 2003; Rena et al., 1999). The expression of liver KLF15 is up-regulated relatively earlier in the time course of fasting, and its expression rapidly decreases during refeeding (Takeuchi et al., 2016; Teshigawara et al., 2005). It is well-known that blood insulin levels also decrease early during fasting and rise steeply during refeeding, consistent with our model that the insulin-FoxO pathway controls *Klf15* gene expression.

Although FoxOs are well known to be involved in gluconeogenesis and lipid metabolism in the liver (Deng et al., 2012; Haeusler et al., 2014; Matsumoto et al., 2006; Nakae et al., 2001; Zhang et al., 2006, 2012), only limited information has been available about the link between FoxOs and amino acid metabolism. In a previous study using Drosophila, it is reported that dFOXO is activated during amino acid starvation and is critical for optimal survival under these conditions (Kramer et al., 2008). It is also reported that the expression of several genes involved in amino acid catabolism is increased in liver-specific FoxO1 transgenic mice (Zhang et al., 2006) and that insulin negatively regulates tyrosine aminotransferase gene promoter activity (Ganss et al., 1994), although the underlying mechanism remained unknown. In accordance with our results, the microarray data GSE60527 of FoxOs KO mouse livers during fasting (Haeusler et al., 2014) showed that FoxOs knockout mice exhibited slight decreases in the hepatic expressions of *Klf15* (0.77 times the control), *Alt1* (0.98), *Prodh* (0.76), *Hpd* (0.96), and *Otc* (0.85) compared to control mice. *Alt1*, *Prodh*, *Hpd,* and *Otc* genes are involved in amino acid metabolism and known to be predominantly regulated by KLF15 in liver (Gray et al., 2007; Jeyaraj et al., 2012b; Takashima et al., 2010). Furthermore, the results of plasma amino acid composition showed that the composition of hepatic FoxO1 and FoxO3a knockdown mice tended to be similar with that of KLF15 knockdown mice (Figure 6N). Thus, our current finding that the function of FoxO is coupled with amino acid metabolism via KLF15 provides better understanding of the regulatory mechanisms of amino acid metabolism by FoxO, which has not been fully elucidated so far (Gross et al., 2008; Teleman, 2009), although our findings do not deny any possible involvement of KLF15-independent pathway(s) in the FoxO regulation of amino acid metabolism.

Regarding lipid metabolism, we have previously reported that KLF15 represses the regulation of *Srebf1c* expression via the nuclear receptor LXR during fasting (Takeuchi et al., 2016). Based on these findings, we demonstrated in the current study that the insulin regulation of *Srebf1c* expression is mediated via the FoxO-KLF15 pathway.

Several papers have been published on the involvement of FoxO in the regulation of lipogenesis and *Srebf1c* expression in the liver (Deng et al., 2012; Kamei et al., 2008; Liu et al., 2010; Matsumoto et al., 2006; Qu et al., 2006; Zhang et al., 2006). Some authors reported that liver-specific FoxO1ADA overexpression represses *Srebf1c* expression and lipogenesis (Deng et al., 2012; Zhang et al., 2006). In addition, FoxO1,3 liver-specific KO mice exhibit enhancement of lipogenesis and *Fasn* gene expression in liver (Zhang et al., 2012), and FoxO1,3,4 liver-specific KO mice show that the lipogenesis in liver is enhanced (Haeusler et al., 2014) and the amount of hepatic triglyceride is increased (Tao et al., 2011), consistent with our results. In contrast, analysis of transient overexpression of constitutively activated FoxO1 in mouse liver using Ad-FoxO1ADA demonstrates that FoxO1 enhances the lipogenesis through SREBP-1c activation (Matsumoto et al., 2006). These increases in lipogenesis result from a feedback loop that enhances insulin signaling, thereby modulating lipid metabolism through SREBP-1c in a FoxO-independent manner. Therefore, the discrepancies could be caused by differences in experimental models or in the duration and level of overexpression, as well as time of assessment with respect to fasting versus feeding (Gross et al., 2008).

The KLF family is evolutionarily conserved in species ranging from Caenorhabditis elegans (C. elegans) to mammalian species including mice and humans. Three KLFs, *klf-1*, *klf-2,* and *klf-3* are present in C. elegans, and have multiple functions, including fat metabolism, autophagy, and cell survival (Brey et al., 2009; Hashmi et al., 2008). Nematode *klf-1* and *klf-3* are involved in extended lifespan because of calorie restriction, and these effects are mediated by *daf-2*, the homolog of the mammalian insulin/IGF receptor (Carrano et al., 2014; Hsieh et al., 2017). Therefore, it is suggested that the insulin-FoxO-KLF15 pathway might be evolutionarily conserved and have influence on divergent biological functions.

During fasting, glucose is supplied into the blood by glycogenolysis as well as gluconeogenesis in the liver using free amino acids produced by proteolysis in muscles and other tissues. By contrast, after a meal, excess glucose is stored in the body in the form of triglycerides through a series of reactions by enzymes involved in lipogenesis. The FoxO-KLF15 pathway is activated during fasting to promote gluconeogenesis from amino acids, and KLF15 inhibits gene expression of the *Srebf1c*, a master regulator of lipogenesis, preventing the conversion of glucose into triglycerides. On the other hand, in the postprandial state, the FoxO-KLF15 pathway is shut down through phosphorylation of FoxOs caused by elevated blood insulin level, thereby stopping gluconeogenesis and promoting lipogenesis simultaneously (Figure 7). Therefore, we could clarify the physiological role of the insulin-FoxO-KLF15 pathway as a mediator of the reciprocal dynamics of gluconeogenesis and lipogenesis. Furthermore, this pathway may be worth noting in skeletal muscles and thereby exert an effect to provide protein-derived carbon for glucose production through effects in multiple tissues.

Overall, these findings demonstrate that the insulin-regulated FoxO-KLF15 axis contributes to the integrated regulation of metabolism between the three macronutrients, protein, carbohydrate, and fat in fasting.

## STAR★Methods

### Key resources table

**Table:** 

REAGENT or RESOURCE	SOURCE	IDENTIFIER
**Antibodies**		

Rabbit anti-FoxO1	Cell Signaling Technology	2880
Rabbit anti-FoxO3a	Cell Signaling Technology	2497
Rabbit anti-Phospho-FoxO1 (Thr24)/FoxO3a (Thr32)	Cell Signaling Technology	9464
Mouse anti-KLF15	Santa Cruz Biotechnology	sc-8035
Mouse anti-LaminA/C	Santa Cruz Biotechnology	sc-376248
Mouse anti-α-Tubulin	Santa Cruz Biotechnology	sc-8035
Rabbit control IgG	Sino biological	CR1

**Bacterial and virus strains**		

Ad-FoxO1ADA6KR	This paper	N/A
Ad-FoxO3AAA	Vector Biolab	1025
Ad-FoxODN	This paper	N/A
Ad-GFP	(Takeuchi et al., 2016)	N/A
Ad-FoxO1,3i	This paper	N/A
Ad-Insri	This paper	N/A
Ad-LacZi	(Takeuchi et al., 2016)	N/A
Ad-KLF15i	(Takeuchi et al., 2016)	N/A
Ad-*Klf15*-Luc (Full)	This paper	N/A
Ad-*Klf15*-Luc (C1)	This paper	N/A
Ad-*Klf15*-Luc (C2) (Ad-*Klf15*-core-Luc)	This paper	N/A
Ad-*Klf15*-Luc (C3)	This paper	N/A
Ad-*Klf15*-Luc (C4)	This paper	N/A
Ad-*Klf15*-Luc (C5)	This paper	N/A
Ad-*Klf15*-Luc (C6)	This paper	N/A
Ad-*Klf15*-Luc (Inverted)	This paper	N/A
Ad-*Klf15*-Luc (Inverted Core)	This paper	N/A
Ad-*Klf15*-Luc (R1mut)	This paper	N/A
Ad-*Klf15*-Luc (R2mut)	This paper	N/A
Ad-*Klf15*-Luc (R1R2mut)	This paper	N/A

**Chemicals, peptides, and recombinant proteins**		

Polypropylene centrifuge tube	Beckman coulter	326823
3.5 ml polypropylene Quick-Seal centrifuge tube	Beckman coulter	349621
ALLN (Protease inhibitor)	Calbiochem	208719
T0901317	Cayman Chemical	71810
40 μm mesh cell strainer	Corning	352340
Dithiothreitol (DTT)	FUJIFILM Wako chemicals	040-29223
D-Luciferin Potassium Salt	FUJIFILM Wako chemicals	126-05116
DMEM containing 25 mM glucose	FUJIFILM Wako chemicals	043-30085
HBSS	FUJIFILM Wako chemicals	084-08345
L-glutamine	FUJIFILM Wako chemicals	073-05391
Penicillin-Streptomycin	FUJIFILM Wako chemicals	168-23191
RPMI1640	FUJIFILM Wako chemicals	189-02025
Sodium pyrophosphate (Phosphatase inhibitor)	FUJIFILM Wako chemicals	529-91151
Spermidine	FUJIFILM Wako chemicals	195-09821
Spermine	FUJIFILM Wako chemicals	198-09811
Streptozotocin	FUJIFILM Wako chemicals	195-15154
PD-10 desalting columns containing Sephadex G-25 resin	GE Healthcare	17085101
Sterile gauze	Kawamoto Corporation	7161
0.45 μm centrifuge filter	Merck	UFC30HV00
Dexamethasone	Nacalai tesque	11107-64
Formaldehyde	Nacalai tesque	16222-65
Proteinase K	Nacalai tesque	15679-06
Phenol-chloroform	Nacalai tesque	26058-54
Sepasol-RNA I	Nacalai tesque	09379-55
Sodium Fluoride (Phosphatase inhibitor)	Nacalai tesque	31420-82
Reporter Lysis Buffer	Promega	E3971
Aprotinin (Protease inhibitor)	Sigma-Aldrich	A3428
Insulin	Sigma-Aldrich	I5500
Leupeptin (Protease inhibitor)	Sigma-Aldrich	L2884
Pepstatin A (Protease inhibitor)	Sigma-Aldrich	P5318
PMSF (Protease inhibitor)	Sigma-Aldrich	P7626
Sulfosalicylic acid	Sigma-Aldrich	390275
William’s E medium	Sigma-Aldrich	W4128
FBS	Thermofisher	10270-106
Lipofectamin 3000	Thermofisher	L3000015
Pikkagene (Firefly luciferase assay reagent)	Toyo Bnet bio	PGL5500
Collagenase II	Worthington	LS004176

**Critical commercial assays**		

Mouse insulin ELISA kit	FUJIFILM Wako chemicals	634-01481
Triglyceride E-test Wako kit	FUJIFILM Wako chemicals	432-40201
KAPA SYBR Fast qPCR kit	NIPPON Genetics	KK4602
Renilla Luciferase Assay System	Promega	E2820
PrimeSTAR Mutagenesis Basal Kit	TAKARA Bio	R046A
ReverTra Ace qPCR RT Master Mix	TOYOBO	FSQ-201
SuperPrep II Cell Lysis & RT Kit for qPCR	TOYOBO	SCQ-401

**Experimental models: Cell lines**		

Fao	ECACC	89042701
HEK293	(Takeuchi et al., 2016)	N/A
HepG2	(Takeuchi et al., 2016)	N/A
Huh7	JCRB Cell Bank	JCRB0403

**Experimental models: Organisms/strains**		

ICR male mice	Japan SLC	N/A
KLF15KO male mice	(Fisch et al., 2007)	N/A

**Oligonucleotides**		

Q-RT PCR primers	See Tables S1 and S2	N/A
ChIP primers	See Table S3	N/A
Primers for construction of *Klf15*-luc plasmid series	See Table S4	N/A

**Recombinant DNA**		

FLAG-Foxo1-ADA6KR (Expression plasmid encoding the constitutive active mouse FoxO1)	Addgene	17561
pAd promoterless plasmid	Thermofisher	V49420
pAd/CMV/V5-DEST plasmid	Thermofisher	V49320
pENTR4-Luc plasmid	(Nishi-Tatsumi et al., 2017)	N/A
pENTR4 plasmid	Thermofisher	11818-010
pENTR/U6 plasmid	Thermofisher	K494500
pRL-SV40	Promega	E2231

**Software and algorithms**		

ImageJ 1.50i	NIH	https://imagej.nih.gov/ij/
JASPAR2020	JASPAR	https://jaspar.genereg.net/
Living Image™ software2.50	PerkinElmer	https://www.perkinelmer.com/product/spectrum-200-living-image-v4series-1-128113
Python3.8.5	Python Software Foundation	https://www.python.org/
QuantStudio™ Design and Analysis Software v1.5.1	Thermofisher	https://www.thermofisher.com/jp/ja/home/global/forms/life-science/quantstudio-3-5-software.html
Scikit-learn0.23.2	scikit-learn.org	https://scikit-learn.org/stable/
Statview Software version5.0	BrainPower	https://www.statview.com/
UCSC Genome Browser on Mouse July 2007 (NCBI37/mm9) Assembly	UCSC	https://genome.ucsc.edu/

### Resource availability

#### Lead contact

Further information and requests for resources and reagents should be directed to and will be fulfilled by the lead contact, Naoya Yahagi, MD., PhD (nyahagi-tky@umin.ac.jp).

#### Materials availability

All requests for resources and reagents should be directed to and will be fulfilled by the Lead Contact, Naoya Yahagi, MD., PhD (nyahagi-tky@umin.ac.jp). This includes selective antibodies, viruses, serum and proteins. All reagents will be made available on request after completion of a Materials Transfer Agreement.

### Experimental models and subject details

#### Animals

Five to seven-week-old ICR male mice were purchased from Japan SLC. KLF15KO mouse was kindly gifted from Prof. Jain MK and genotyped as previously described (Fisch et al., 2007). All animals were maintained in a temperature-controlled environment with a 14-h-light / 10-h-dark cycle and were given free access to standard laboratory diet and water. For the fasting group, animals were starved 24-h, and for the refeeding group, they were re-fed for 16-h after a 24-h starvation. For the experiments of insulin-depleted diabetic mice, mice were administrated with streptozotocin (two intraperitoneal injections of 100 mg/kg body weight with 1-day interval) as previously described (Takeuchi et al., 2007). Plasma insulin levels were measured using mouse insulin ELISA KIT (Fujifilm). Liver triglyceride levels were measured using triglyceride E-test Wako kit (Fujifilm) as described previously (Yahagi et al., 1999). Mice were sacrificed in the early light phase in a fasted, re-fed state. All animals studied were anesthetized and euthanized according to the protocol approved by the Tsukuba University Animal Care and Use Committee. All experiments were repeated at least twice.

### Method details

#### Measurement of plasma amino acids

To remove the protein components in the collected plasma samples, equal amounts of 3% sulfosalicylic acid were mixed with each sample and the supernatant was collected after centrifugation. The sample pH was adjusted to pH 2-3 and then filtered using a centrifuge filter (Merck). The whole process was operated on ice. The amino acid composition was measured using JLC-500/V2 Automatic Amino Acid Analyzer (JEOL).

#### *In vivo* imaging of luciferase activity

*In vivo* imaging was performed as described previously (Murayama et al., 2019; Takeuchi et al., 2010, 2016). Four days after the adenovirus transduction, animals were starved for 24-h from the early dark phase, and then they were re-fed for 16-h after the starvation. At each nutritional condition, D-Luciferin potassium salt (Fujifilm) was injected i.p. into mice and the luminescence in the liver was captured using an IVIS™ Imaging System (PerkinElmer). Relative photon emission over the liver region was quantified using Living Image™ software (PerkinElmer). Two paired data from the same animal on the different nutritional conditions (i.e., fasted or re-fed) was continuously obtained and the ratio between the two quantities was used to cancel the variations in hepatic transduction efficiencies. Results less than 1 x 10^5^ counts/min (2.44 x 10^6^ photons) on both the conditions were not adopted due to inadequate detection accuracy.

#### Quantitative reverse transcription PCR (Q-RT PCR)

Total RNA (500 ng) was reverse-transcribed using the ReverTra Ace qPCR RT Master Mix (TOYOBO). Sample preparation for Q-RT PCR from culture cells was performed using the SuperPrep II Cell Lysis & RT Kit for qPCR (TOYOBO). Q-RT PCR was performed using SYBR Green Dye (NIPPON Genetics) on a QuantStudio™ 5 Real-Time PCR System (Thermofisher) and quantified by standard curve method with cDNA as template. After amplification by PCR, samples containing the product with the correct Tm value were adopted using the melting curve plot for each sample. To calculate mRNA copy number of each *Klf15* variants, plasmids containing each gene were used as template for preparing the standard curve. The primer sets are listed in Tables S1 and S2. As the correction of the gene expression level for each sample, *Rplp0* was used in Figures 3G, 3H, 4I–4L, S1B, S1H, and S3A; *Gapdh* was used in Figures 3A, 3B, 3D, 3E, 3J, 3K, 6A, 6B, S3B, and S6C–S6F; *Actb* was used in Figures 4A–4C, 4E–4H, and 4M–4P; *Cyclophilin B* was used in Figures S6A and S6B; *rCyclophilin A* as used in Figures 2M, 2N, and S2D; and *CYCLOPHILIN A* was used in Figures 2A–2J.

#### Chromatin immunoprecipitation (ChIP) assay

ChIP assays using mouse liver was performed as described previously (Nakagawa et al., 2016; Takeuchi et al., 2016). Briefly, liver tissue samples from the fasted and re-fed mice were minced in PBS and cross-linked in 1.5% formaldehyde for 15 min at room temperature. Fixed samples were homogenized and then subjected to sonication for DNA fragmentation. After centrifugation, supernatant was diluted and then subjected to immunoprecipitation with anti-FoxO1 or with control IgG bound to Dynabeads magnetic beads (Thermofisher) and rotated overnight at 4°C. The complexes were washed and then incubated overnight at 65°C for reverse crosslinking. DNA-protein complex was treated with Proteinase K and chromatin DNA was purified with phenol-chloroform, eluted in TE buffer and subjected to Q-PCR analysis. The primer sets are listed in Table S3.

#### Immunoblotting

Immunoblotting was performed as described previously (Takeuchi et al., 2010, 2016). Nuclear extract protein from mouse liver was prepared as previously described (Sheng et al., 1995; Yahagi et al., 2003, 2004). Liver tissues (1.5 g) collected from 3-4 mice were pooled and homogenized in 15 ml of buffer A (10 mM Hepes at pH 7.6, 25 mM KCl, 1 mM EDTA, 2 M sucrose, 10 % glycerol, 0.15 mM spermine, 2 mM spermidine, protease inhibitors and phosphatase inhibitors). The sample was filterd with sterile gauze (Kawamoto Corporation) and layered on 15 ml of buffer A in a polypropylene centrifuge tube (Beckman coulter). The tube was centrifuged at 24,000 r.p.m. for 90 min at 4°C. The pellet was suspended in 800 μl of buffer B (10 mM Hepes at pH 7.6, 100 mM KCl, 2 mM MgCl_2_, 1 mM EDTA, 1 mM DTT, 10% glycerol, protease inhibitors and phosphatase inhibitors) and centrifuged at 89,000 r.p.m. for 20 min at 4°C. The supernatant was used as a nuclear extract. For total cell lysates from culture cells, cells were harvested and resuspended in lysis buffer (50 mM Tris-HCl at pH 7.5, 137 1 mM EDTA, 1% Triton-X, protease inhibitors and phosphstase inhibitors). Lysates were incubated on ice for 30 min and centrifuged at 15000 r.p.m. for 10 min. The supernatant was used as a total cell lysate.

#### RNA isolation and RNA-seq analysis

RNA preparation from mouse liver was performed as previously described (Takeuchi et al., 2010). For preparation of RNA from liver of fasted mice, liver tissues collected from 3 mice were pooled and lysed with Sepasol-RNA I (Nacalai tesque) and RNA was extracted according to manufacture protocol. RNA quality was checked by Agilent 2100 BioAnalyzer and then RNA-seq analysis using RNA were performed with Illumina Hiseq 2500 by outsourcing to TAKARA Bio Inc. The RNA-seq data have been deposited to DDBJ database (accession number: DRA013049).

#### Preparation and transduction of recombinant adenoviruses

The various DNA fragments of mouse *Klf15* promoter region were amplified by PCR using mouse genomic DNA as template and inserted into multiple cloning site on the pENTR4-Luc Gateway entry vector linked to firefly luciferase reporter (*Klf15*-Luc) as previously described (Nishi-Tatsumi et al., 2017). Mutated *Klf15*-core-luc plasmids were generated using PrimeSTAR Mutagenesis Basal Kit (Takara Bio) based on PCR. The primer sets for PCR and brief methods are listed in Table S4. Adenoviral constructs were generated by homologous recombination between the entry vector and the pAd promoterless vector (Thermofisher). To construct dominant-negative form FoxO1/3/4 (FoxODN), FoxO1 cDNA fragment lacking of transactivation domain from C-terminus was amplified by PCR using TFEL FoxO1 plasmids as template. The DNA fragment encoding FoxO1ADA6KR and FoxODN were subcloned into pENTR4 and adenovirus encoding FoxO1ADA6KR and FoxODN (Ad-FoxO1ADA6KR and Ad-FoxODN respectively) were generated by homologous recombination between the entry vector and the pAd/CMV/V5-DEST vector (Thermofisher). Adenovirus encoding GFP (Ad-GFP) was described previously (Takeuchi et al., 2016). Briefly, GFP gene inserted in the entry vector was homologously recombined with the adenovirus vector. The mouse FoxO1 and FoxO3a-specific shRNA construct (FoxO1,3i), and the mouse insulin receptor-specific shRNA construct (Insri) were subcloned into pENTR/U6 entry vector (Thermofisher) using these sequences: 5’- GATCTACGAGTGGATGGTG -3’ and 5’- CGGTTAAGACTGTCAATGA -3’, followed by homologous recombination with the pAd promoterless vector (Ad-FoxO1,3i and Ad-Insri respectively). Adenovirus encoding LacZ-specific shRNA for RNAi (LacZi) was described previously (Takeuchi et al., 2016). Briefly, LacZ-specific shRNA construct inserted in pENTR/U6 the entry vector was homologously recombined with the adenovirus vector. Recombinant adenoviruses were propagated in HEK293 cells and purified by CsCl gradient centrifugation as described previously (Takeuchi et al., 2007). Solution 1 (5.30g of CsCl in 8.7 ml of 10 mM Tris-HCl at pH 7.9) and Solution 2 (2.68g of CsCl in 9.2 ml of 10 mM Tris-HCl at pH 7.9) were prepared, and first 1.02 ml of solution 1, then 0.78 ml solution 2 were layered in 3.5 ml polypropylene Quick-Seal centrifuge tube (Beckman Coulter). The cell lysate containing recombinant adenovirus was gently overlaid and centrifuged for 3 hr at 89,000 r.p.m. at 4°C. The tube was punctured at the bottom with a 21-gauge needle, and fraction containing adenovirus was collected. To further purify recombinant adenovirus, the collected sample was fractionated on a PD-10 desalting columns containing Sephadex G-25 resin (GE Healthcare) by adding phosphate-buffered saline (PBS) with 1mM MgCl_2_. For animal experiments, adenoviruses were injected intravenously into male mice from subclavian vein at the following doses: for *Klf15*-Luc (C1, C2, C4, C5, Ex2, Inverted, Inverted Core, R1mut, R2mut and R1R2mut), 4 x 10^7^ P.F.U.; for *Klf15*-Luc (Full, C3 and C6), 2 x 10^8^ P.F.U.; for FoxO1ADA6KR, 2 x 10^8^ P.F.U.; for FoxO3aAAA, 5 x 10^8^ P.F.U.; for FoxODN, 5 x 10^8^ P.F.U.; for FoxO1,3i, 7.5 x 10^8^ P.F.U.; for Insri,1.25 x 10^9^ P.F.U., and 1000 optical particles of adenovirus were calculated as 1 P.F.U. For culture cell experiments, adenoviruses were infected at the following m.o.i.: in HepG2 and Huh7, 10 m.o.i.; in Fao, 7 m.o.i.; in primary hepatocytes, 0.3 m.o.i., and these titers were calculated with Karber equation.

#### Isolation and culture of primary hepatocytes

Primary hepatocytes were isolated from WT and KLF15KO mouse with collagenase perfusion method. A mouse was anesthetized, and the portal vein was cannulated with a 26-gauge needle. HBSS containing 0.5 mM EDTA was perfused to chelate calcium, and then HBSS containing 5 mM CaCl_2_ and 1 mg/ml collagenase II was perfused to dissociate extracellular matrix of the liver. After the liver dissection, cells were filtered with 40 μm mesh cell strainer, and hepatocytes were purified by gradient centrifugation method. Hepatocytes were suspended in DMEM containing 25 mM glucose, 100 nM insulin, 10 nM dexamethasone, 100 U/ml penicillin, and 100 μg/ml streptomycin sulfate supplemented with 10% FBS and plated at 2 x 10^4^ cells/cm^2^ for 3h. For adenovirus infection, the medium was changed to William’s E medium containing the indicated adenoviruses, and 2 mM L-Glutamine, 100 U/ml penicillin, and 100 μg/ml streptomycin sulfate supplemented with 5% FBS and 10 μM T0901317 for 24-h, then replaced to the medium without adenoviruses for 24-h.

#### Cell culture

HEK293 human embryonic kidney cells, HepG2 and Huh7 human hepatoma cells were cultured in DMEM containing 25 mM glucose, 100 U/ml penicillin, and 100 μg/ml streptomycin sulfate supplemented with 10% FBS. Fao rat hepatoma cells were cultured in RPMI1640, 100 U/ml penicillin, and 100 μg/ml streptomycin sulfate supplemented with 10% FBS. For insulin stimulation on Fao cells, the cells were starved in serum-free medium containing 0.01 nM insulin and 10 nM dexamethasone for 24-h, and then the medium was changed to be treated with 100 nM insulin for 24-h as described previously (Sano et al., 2016; Yugi et al., 2014).

#### Luciferase assay and TFEL scan genome-wide transcription factor screening

For luciferase assay, HepG2 cells were seeded in a 48well plate and incubated to 10-20% confluent. The indicated amounts of expression plasmids, firefly luciferase reporter plasmid and Renilla luciferase reporter plasmid (pRL-SV40 (Promega)) were co-transfected into cells using Lipofectamin3000 reagent (Thermofisher) according to the manufacturer’s protocol. Total amounts of transfected DNA were adjusted with empty plasmid. The luciferase activity in transfectants was measured as described previously (Takeuchi et al., 2016). Cells were suspended in 100 μl of Reporter Lysis Buffer (Promega) and centrifuged at 15000 r.p.m. for 15 min at 4°C. The supernatant was mixed with Pikkagene reagent (Toyo Bnet bio) and the firefly luciferase activity was measured using a Wallac ARVO SX 1420 luminometer (PerkinElmer). Renilla luciferase activity was measured with Renilla Luciferase Assay System (Promega) according to the manufacturer protocol. The renilla luciferase activities were used to normalize transfection efficiencies. The transcription factor expression library (TFEL) clones (Yahagi and Takeuchi, 2021) were co-transfected with the *Klf15*-core-luc plasmid into HepG2 cells. The luciferase activity in transfectants was then measured with the method described above.

#### Statistical analyses

Data are expressed as means ± s.e.m. Differences between two groups were assessed using the unpaired two-tailed Student’s t-test. Data sets involving more than two groups were assessed by ANOVA with Statview Software (BrainPower). The differences were considered to be significant if *P* < 0.05. (∗*P* < 0.05 and ∗∗*P* < 0.01).

## Data Availability

The RNA-seq data have been deposited to DDBJ database (accession number: DRA013049). All data and code are available from the corresponding authors upon reasonable request.
