# Supplementary material for: FoxO-KLF15 pathway switches the flow of macronutrients under the control of insulin
Source: iScience. 2021 Nov 15;24(12):103446. doi: 10.1016/j.isci.2021.103446 (PMC8710527; doi:10.1016/j.isci.2021.103446)
Supplement: Document S1. Figures S1–S6 and Tables S1–S4 [file mmc1.pdf]

iScience, Volume *24*

Supplemental information

FoxO-KLF15 pathway switches
the flow of macronutrients
under the control of insulin

Yoshinori Takeuchi, Naoya Yahagi, Yuichi Aita, Zahra Mehrazad-Saber, Man Hei Ho, Yiren Huyan, Yuki Murayama, Akito Shikama, Yukari Masuda, Yoshihiko Izumida, Takafumi Miyamoto, Takashi Matsuzaka, Yasushi Kawakami, and Hitoshi Shimano

Supplemental information

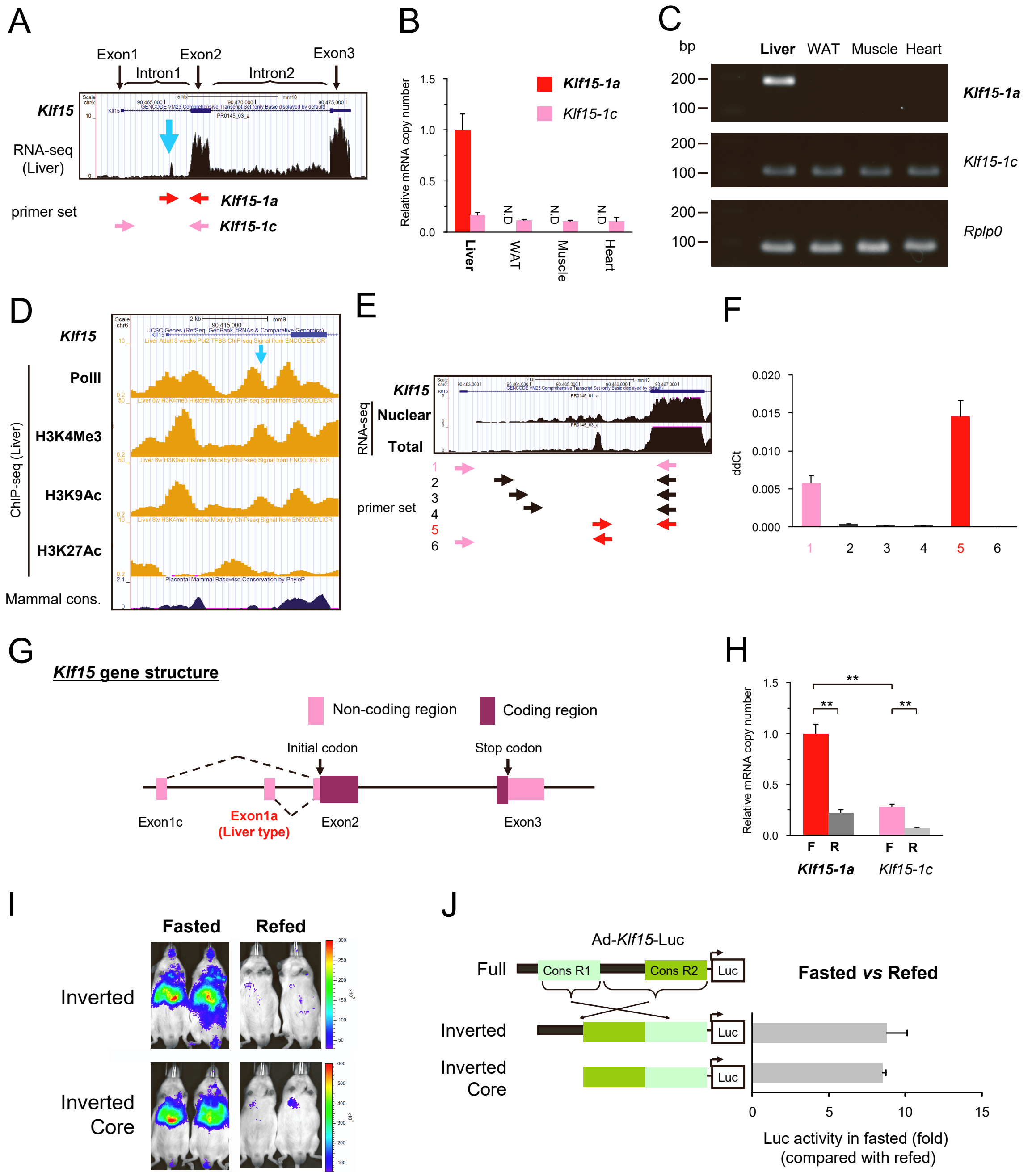

Figure S1. Identification of liver-specific klf15 variant and its regulatory genomic regions by fasting, related to Figure 1.

(A) Identification of liver type exon (Ex1a) on *Klf15* gene from RNA-seq analysis of liver in the fasted state. Ex1a peak is indicated by a light blue arrow. Each red and pink arrows mean Q-RT PCR primer sets for detection of conventional *Klf15* (*Klf15-1c*) and liver type *Klf15* (*Klf15-1a*) variant respectively.

(B and C) *Klf15-1a* variant is expressed highly in liver (n = 3). Results of Q-RT PCR analysis (b) and electrophoresis (c) with the Q-RT PCR products.

(D) RNA polII binding and modified Histones binding regions on *Klf15* gene from ENCODE ChIP-seq database (Liver 8w by ChIP-seq Signal from ENCODE/LICR) on Genome browser. These peaks means promoter/enhancer regions. Mammalian conservation regions are shown below ChIP-seq peaks data. The light blue arrow indicates the location of Ex1a.

(E and F) Estimation of *Klf15-1a* variant TSS from RNA-seq results with nuclear RNA of liver in fasted state and Q-RT PCR analysis with various primer sets (n = 6).

(G) Schema of *Klf15* gene structure.

(H) Q-RT PCR analysis of *Klf15-1a* and *1c* variants using liver RNA samples in fasted and re-fed states. *Klf15-1a* expression increases in fasted state same as conventional *Klf15-1c* (n = 3). F, fasted; R, re-fed.

(I and J) in vivo Ad-luc promoter analyses of the *Klf15-1a* gene. Images (I) and hepatic luciferase activities (J) of mice injected with the indicated Ad-*Klf15*-Luc are shown (n = 5–6).

Data sets were assessed by ANOVA. The differences were considered to be significant if $P < 0.05$.

(* $P < 0.05$ and ** $P < 0.01$)

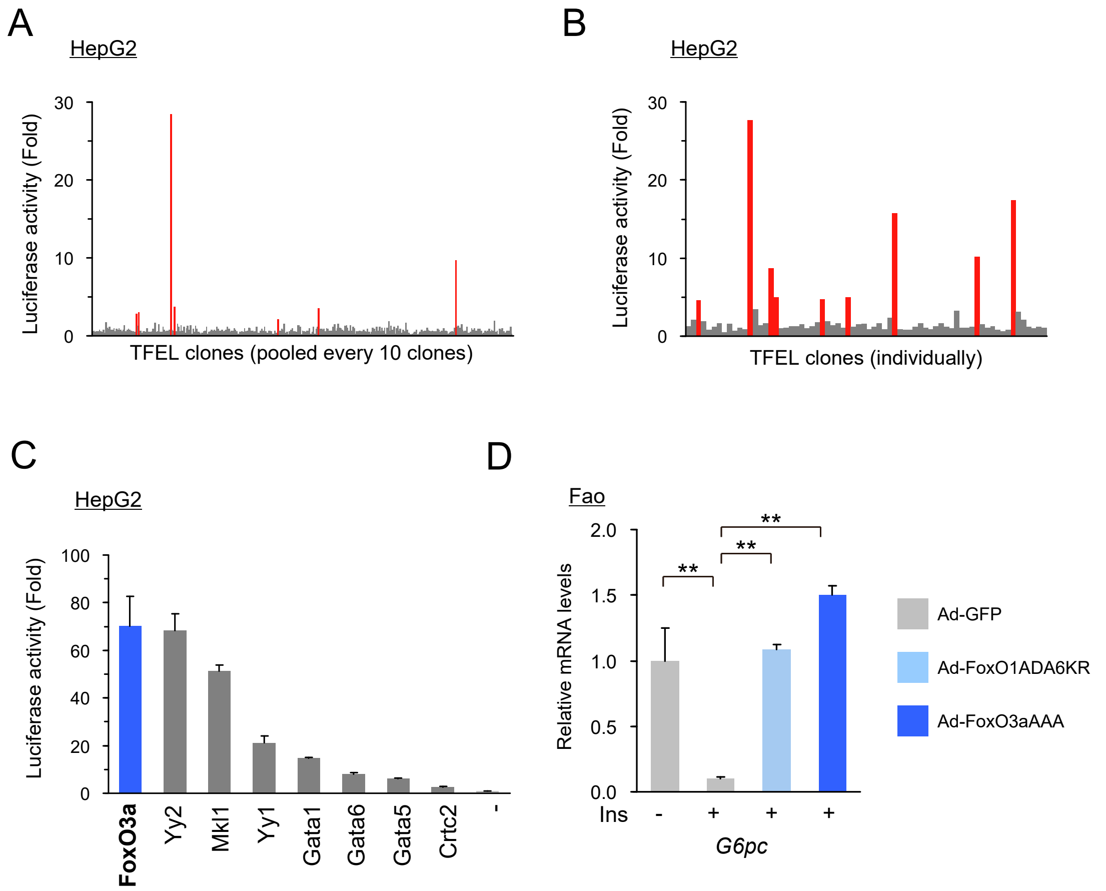

Figure S2. Identification of FoxO1 and FoxO3a on *Klf15* expression in hepatocytes, related to Figure 2.

(A) Result of *Klf15*-core-luc in 1st TFEL screening. TFEL plasmids pooled every 10 clones were co-transfected with *Klf15*-core-luc in HepG2. The cutoff value is 2 and 7 pooled samples (shown as red bar) were proceed to 2nd TFEL screening for evaluation individually.

(B) Result in 2st TFEL screening. Each TFEL plasmids were co-transfected with *Klf15*-core-luc in HepG2. Eight clones (shown as red bar) were proceed to 3rd TFEL screening for confirmation. One of the 9 red bar was duplicated.

(C) Candidate trans-activator of *Klf15*-core-luc on HepG2 in 3rd TFEL screening (n = 3). The indicated gene expression plasmids from TFEL were co-transfected with *Klf15*-core-luc in HepG2.

(D) Rescue exprerimennts of FoxO1 and FoxO3a on *Klf15* suppression by insulin in Fao rat hepatoma cell lines. The cells were starved in serum-free medium containing 0.01 nM insulin and 10 nM dexamethasone for 2-h, and then the medium was changed to be treated with 100 nM insulin for 24-h. Q-RT PCR analysis of *G6pc* genes as FoxO target in insulin treated Fao cells. FoxO1ADA6KR and FoxO3aAAA were over-expressed in Fao cells using adenoviruses for 24-h before insulin treatment (n = 5).

Data sets were assessed by ANOVA. The differences were considered to be significant if $P < 0.05$. (*$P < 0.05$ and **$P < 0.01$)

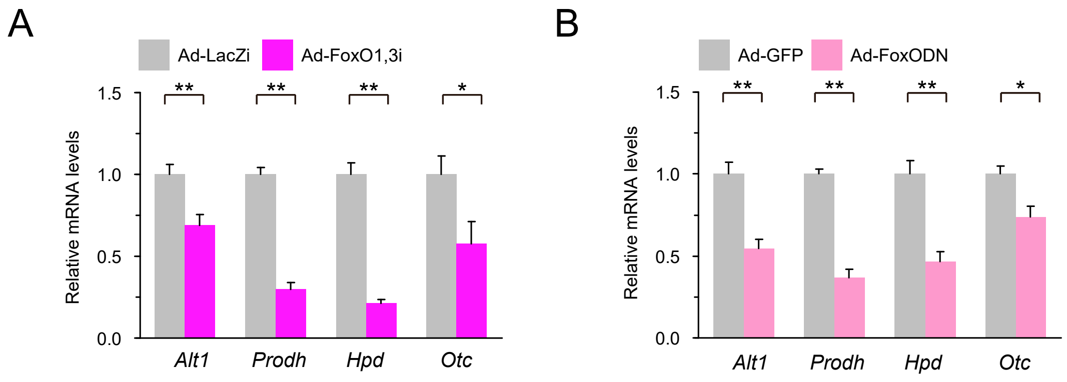

Figure S3. Expression levels of KLF15 target genes by suppression of FoxOs, related to Figure 3.

(A and B) Q-RT PCR analysis of the indicated genes on hepatic FoxO1 and FoxO3a knocked-down liver RNA samples and flag-tagged dominant-negative (DN) FoxO1 protein expressed liver RNA samples in fasted mice (n = 6). 3-days (A) and 5-days (B) after the transduction of each adenovirus, mice were starved for 24-h from the light phase. *Alt1*, *Prodh*, *Hpd*, and *Otc* are known as the genes regulated by KLF15 in liver.

Data were assessed using the unpaired two-tailed Student's t-test. The differences were considered to be significant if $P < 0.05$. ($^*P < 0.05$ and $^{**}P < 0.01$)

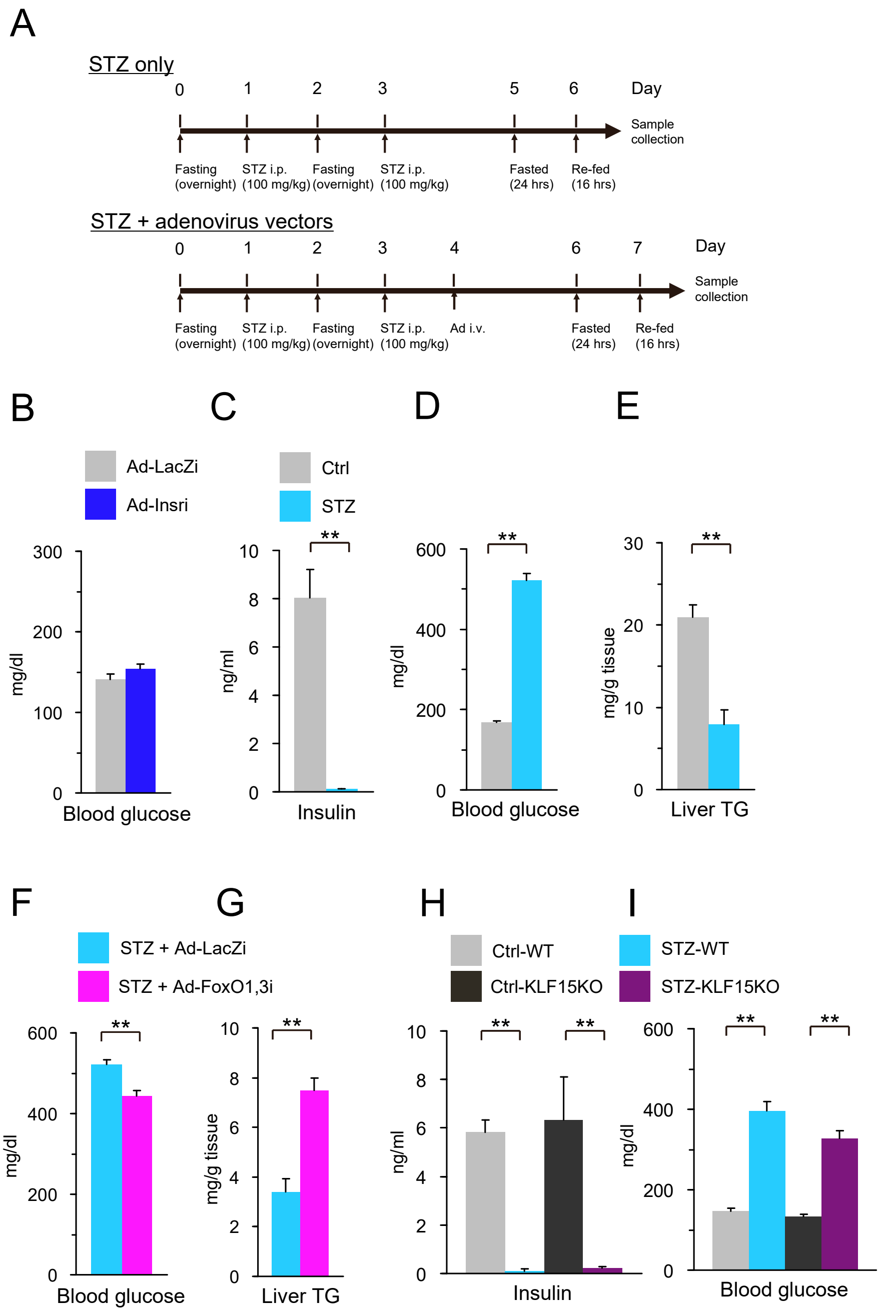

Figure S4. Metabolic parameters under each condition, related to Figure 4.

(A) The experimental procedure for STZ treated mice. Blood glucose level from the tail veil was measured on day4 and the last day.

(B) Blood glucose levels for the mice under the condition by the procedure described in Figure 4

A-C (n = 8).

(C-E) Plasma insulin (C), blood glucose (D) and liver triglyceride (E) levels for the mice under the condition by the procedure described in Figure 4 E-H (n = 9).

(F and G) Blood glucose (F) and liver triglyceride (G) levels for the mice under the condition by the procedure described in Figure 4 I-L (n = 9-10).

(H and I) Plasma insulin (H) and blood glucose (I) levels for the mice under the condition by the procedure described in Figure 4 M-P (n = 8-11).

Differences between two groups were assessed using the unpaired two-tailed Student's t-test. Data sets involving more than two groups were assessed by ANOVA. The differences were considered to be significant if $P < 0.05$. (* $P < 0.05$ and ** $P < 0.01$)

A

Cons R1

```
CCCCTGGAGGGCACAGGGGAGGTTTGCTACCACGGTATGGGAAGGTGAAA
TCTCAAATGTTTGACTGAGTCCTTCAGATATTGCCCCCGACCATGGGGAG
TCCCCGCCGCCCTACAGGTGCGAGAATGGCAGTAACTTTGGGGCCTTCGG
ACTCCTCAGGGAGAAGACTCTTATTTTGAAGACCGGCCCCCATTCGCCAC
CCCCGTGATTTGCACGCTGACCCAATGGCTAGCCCTTGGCCGGCTCTCGG
CTTGTTATCAATTACATGTTGTTCCTACAGCCGCTGTGTCCCCGGGAGGA
TAAACAGCAGGCCTGGCCGGCTAAGAGAGGGGGCGGAGTCCAGCGGCCTG
CCGGGGACCGTGCCCAGAACAGGGTGCCCCCTCCCCCTGCACGCAGCCTG
CGCCTCTGGCTACGCCACGTGCTGCTGTTTTTCTGGGACCGGCTTGATCG
CTGGCACTT
```

B

Cons R2

```
TTCGGCAGCTGGTCAGTACCGGGTGCTAGGCTGTAGAGTGGACCTGAAGT
CAGGTGGACAGGATGCAGCCTGAGCATCCTCCCCATCAGTTTGATAATCA
CACAGGCAGACATGGTCACTGGGCGGGACAAAAGTCCAGAGAGGTGGCCC
CATCACAGCCAGAGACTGTTGACTCTTCTTGGATACCAGACCTAGTATGG
CCTTTGGCTTCAGAGGACACAGCTTTCCCTTTTCCTTCCGATCCCCGCTC
TCTGAGCCCCTCTTGGATACCTGGTGTGGTGCTGAGTGTTGGGGACCCAG
GAGAGTGTGCTGCCAAGAAGGGACACAGGCATCTGCTGAGCCCCCCAAGT
CGGTGAGTGTCCCAGACAGGAGAGCTGGGCTGGGGAGGCAGAACAGGAGG
C
```

C

Cons R1

Matrix ID	Name	Score	Relative score	Strand	Predicted sequence
**MA0480.1**	**Foxo1**	**13.2489**	**0.943951**	**-**	**tgctgtttatc**
MA0480.1	Foxo1	11.26	0.913599	+	tgctgttttc
MA0157.1	FOXO3	9.02966	0.89545	+	gataaaca
MA0157.1	FOXO3	7.68602	0.850314	-	gaaaaaca
MA0157.1	FOXO3	6.85145	0.822279	-	tgataaca
MA0480.1	Foxo1	5.2517	0.821913	+	gcttgttatca
MA0157.1	FOXO3	6.40607	0.807318	-	gggacaca
MA0480.1	Foxo1	3.88244	0.801018	+	acatgttgttc

D

Cons R2

Matrix ID	Name	Score	Relative score	Strand	Predicted sequence
**MA0480.1**	**Foxo1**	**8.97696**	**0.878761**	**+**	**gactgttgact**
MA0157.1	FOXO3	6.40607	0.807318	+	gggacaca

E

Cons R1

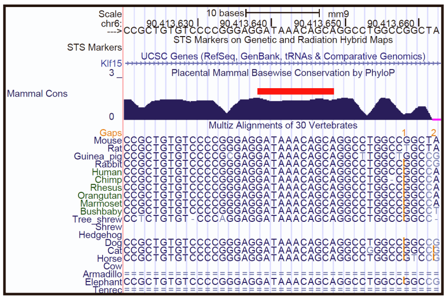

F

Cons R2

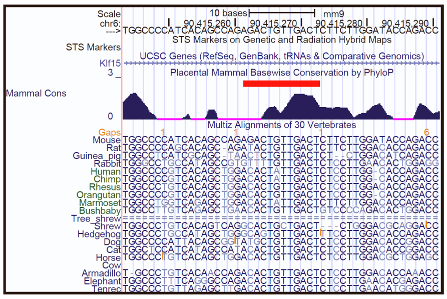

Figure S5. Genomic information on *Klf15-1a* regulatory region, related to Figure 5.

(A-D) Mouse genomic sequence of ConsR1 (A) and ConsR2 (B) inserted in *Klf15*-core-luc. Estimation of FoxOs binding sites on ConsR1 (C) and ConsR2 (D) by JASPAR2020

(http://jaspar.genereg.net/). The highest-scoring sequences in each region were underlined in red (A and B) and mutated for the experiments in Figure 5 B-D.

(E and F) Comparison of genomic sequences between various mammalian species regarding FoxOs binding sites on *Klf15-1a* regulatory region ConsR1(E) and ConsR2(F) by “Placental Mammal Basewise Conservation by PhyloP” on Genome browser. The sequences under red line show the FoxO binding sites. Each FoxO binding site is conserved highly among mammalian species.

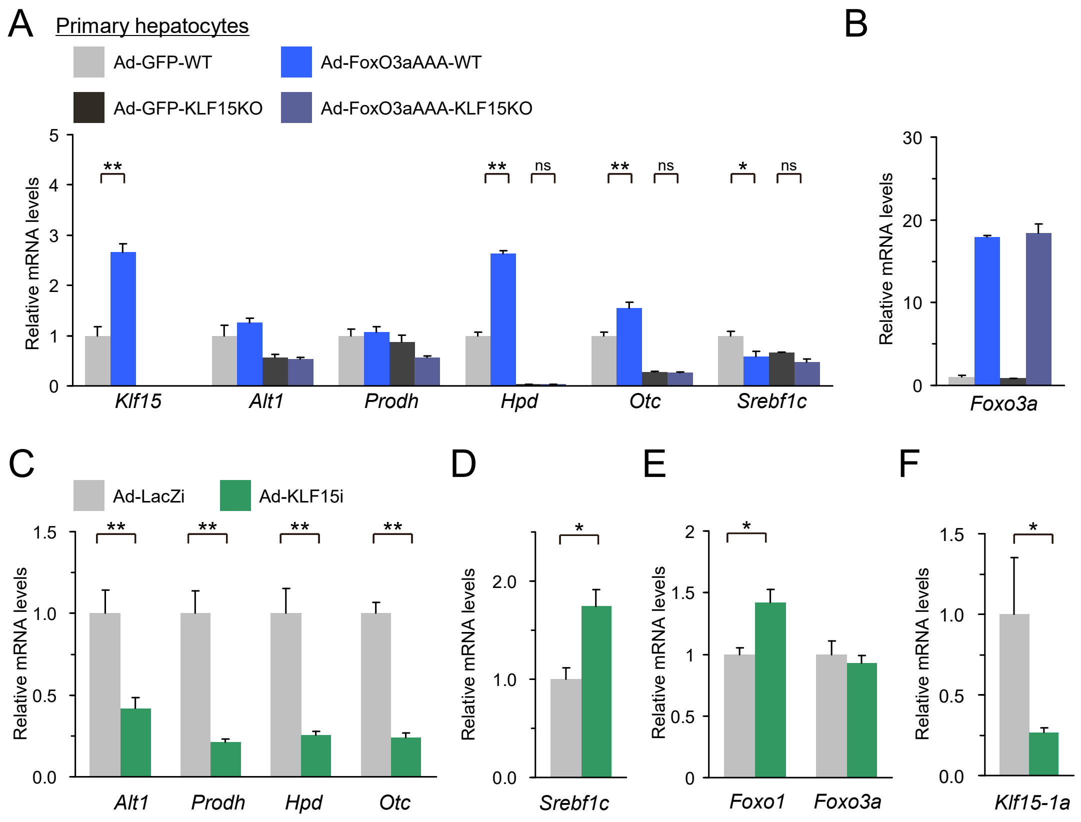

Figure S6. Effects of FoxO3a over-expression and *Klf15* disruption on amino acid metabolism genes, related to Figure 6.

(A and B) Q-RT PCR analysis *Klf15* and the target genes in primary hepatocyte RNA samples from WT and KLF15KO mice (n = 3). Constitutive active FoxO3a was over-expressed in cells using Ad-FoxO3aAAA for 48-h.

(C-F) Q-RT PCR analysis of liver RNA samples from hepatic *Klf15* knocked-down mice using Ad-KLF15i in fasted state (n = 5-6). Four days after the adenovirus transduction, mice were starved for 24-h from the light phase.

Differences between two groups were assessed using the unpaired two-tailed Student's t-test. Data

sets involving more than two groups were assessed by ANOVA. The differences were considered to be significant if $P < 0.05$. (*$P < 0.05$ and **$P < 0.01$)

SUPPLEMENTAL TABLE

Table S1. List of primer sets used for Q-RT PCR.

Gene (mouse)	sequences for Q-RT PCR primers
Klf15-1c	5'-CAGGCGACGCGGAGT-3'<br>5'-CCCTAGGTAACCCACTGAGC-3'
Klf15-1a	5'-CTGGTGTGGTGCTGAGTGT-3'<br>5'-CCCTAGGTAACCCACTGAGC-3'
Klf15	5'-GCGAGAAGCCCTTTGCCT-3'<br>5'-GCTTCACACCCGAGTGAGAT-3'
Foxo1	5'-TTCAATTCGCCACAATCTGTCC-3'<br>5'-GGGTGATTTTCCGCTCTTGC-3'
Foxo3a	5'-TGTGCCCTACTTCAAGGATAAG-3'<br>5'-TTCATTCTGAACGCGCAT-3'
Pck1	5'-TGTCATCCGCAAGCTGAAGA-3'<br>5'-TTCGATCCTGGCCACATCTC-3'
Srebf1c	5'-CGGCGCGGAAGCTGT-3'<br>5'-TGCAATCCATGGCTCCGT-3'
Alt1	5'-TGTCATCCGCAAGCTGAAGA-3'<br>5'-TTCGATCCTGGCCACATCTC-3'
Prodh	5'-CTTCACCAACACGCAGGA-3'<br>5'-ATCTCTTTGCGCTCCGC-3'
Hpd	5'-CATTTCCACTCGGTGACCT-3'<br>5'-TGTCTTGCTCCACCCATG-3'
Otc	5'-AACACGAGCTATAAGAACTCA-3'<br>5'-CAACTGAAGAAGAGTGAC-3'
Gapdh	5'-TGTGTCCGTCGTGGATCTGA-3'<br>5'-CACCACCTTCTTGATGTCATCATAC-3'
Actb	5'-AGCCATGTACGTAGCCATCCA-3'<br>5'-TCTCCGGAGTCCATCACAATG-3'
Rplp0	5'-CCTGAAGTGCTCGACATCACA-3'<br>5'-GCGCTTGTACCCATTGATGA-3'
Cyclophilin B	5'-TGGCTCACAGTTCTTCATAACCA-3'<br>5'-ATGACATCCTTCAGTGGCTTGTC-3'

Gene (rat)	sequences for Q-RT PCR primers
rKlf15	5’-GCGAGAAGCCCTTTGCCT-3’<br>5’-GCTTCACACCCGAGTGAGAT-3’
rG6pc	5’-ACATTCAAGAGACTGTGGGC-3’<br>5’-GGAGCTGTTGCTGTAATAGTCAG-3’
rSrebf1c	5’-GACGCTGTAGGGGTAGCGT-3’<br>5’-TGCAATCCATGGCTCCGT-3’
rCyclophilin A	5’-TTCTTCGACATCACGGCTG-3’<br>5’-TTGCCACCAGTGCCATTATG-3’
GENE (human)	sequences for Q-RT PCR primers
FOXO3a	5’-GTGCCCTACTTCAAGGATAAGGGC-3’<br>5’-ACAGGTTGTGCCGGATGGAG-3’
KLF15	5’-ACACGGGTGAGAAGCCCTTC-3’<br>5’-CTGGTACGGCTTCACACCTG -3’
G6PC	5’-TGGAGACTGGCTCAACCTC-3’<br>5’-CTGGTCCAGTCTCACAGGTTAC-3’
SREBF1c	5’-GCGCCTTGACAGGTGAAGTC-3’<br>5’-CATGGCTCCGCGATCTG-3’
CYCLOPHILIN A	5’-GCATACGGGTCCTGGCATCTTGTCC-3’<br>5’-ATGGTGATCTTCTTGCTGGTCTTGC-3’

Table S2. List of primer sets used for Q-RT PCR in Figure S1 E.

Primer set	sequences for Q-RT PCR primers
1	5’-CAGGCGACGCGGAGT-3’<br>5’-CCCTAGGTAACCCACTGAGC-3’
2	5’-ATGCCATTTGCCTGAGGT -3’<br>5’-CCCTAGGTAACCCACTGAGC-3’
3	5’-TTACTCCTGGATTCTGGAAAC-3’<br>5’-CCCTAGGTAACCCACTGAGC-3’
4	5’-TTTTGACTCTAGATCTAGCCCTGTAG-3’<br>5’-CCCTAGGTAACCCACTGAGC-3’
5	5’-CTGGTGTGGTGCTGAGTGT-3’<br>5’-CCCTAGGTAACCCACTGAGC-3’
6	5’-CAGGCGACGCGGAGT-3’<br>5’-GCTCAGTGGGTTACCTAGGG-3’

Table S3. List of primer sets used for ChIP assay.

Primer set	sequences for ChIP primers
1	5’-GCTCCCAGAGACTAGGCATTC-3’<br>5’-GCTTCCAACTCCCAGGATAC-3’
2	5’-ACCCAATGGCTAGCCCTTG-3’<br>5’-AAGTGCCAGCGATCAAGCC-3’
3	5’-TCAGGTGGACAGGATGCAG-3’<br>5’-CACCACACCAGGTATCCAAGAG-3’

Table S4. List of primer sets and methods used for construction of Klf15-luc plasmid series.

Mutagenesis PCR was performed with PrimeSTAR Mutagenesis basal kit (Takara Bio Inc.)

Klf15-luc	Inserted fragments	Primer sequences
Full	PCR product using the following primers	5’- ACGCGTCTTGGGGATCACGGGC-3’<br>5’- AAGCTTGCCTCCTGTTCTGCCTCC-3’
C1	PCR product using the following primers and C6 PCR product	5’- GAGCTCCTTGGGGATCACGGGC-3’<br>5’- ACGCGTAAGTGCCAGCGATCAAGC-3’
C2	C5 and C6 PCR products	-
C5	PCR product using the following primers	5’-GAGCTCCCCCTGGAGGGCACAG-3’<br>5’-ACGCGTAAGTGCCAGCGATCAAGC-3’
C6	PCR product using the following primers	5’-ACGCGTTTCGGCAGCTGGTCAGTA-3’<br>5’-AGATCTGCCTCCTGTTCTGCCTCC-3’
	Mutagenesis PCR	Primer sequences
C3	PCR product using the following primers and C2 as a template	5’-CCGAGCTCGGGAGGATAAACAGCAGG-3’<br>5’-TCCTCCCGAGCTCGGTACCTATCGA-3’
C4	PCR product using the following primers and C2 as a template	5’-TTACGCGTCTTCAGAGGACACAGCTT-3’<br>5’-TCTGAAGACGCGTAAGTGCCAGCGA-3’
R1mut	PCR product using the following primers and C2 as a template	5’- GAGGAGCGCCAGCAGGCCTGGCCGGCT-3’<br>5’- CTGCTGGCGCTCCTCCCGGGGACACAGC-3’
R2mut	PCR product using the following primers and C2 as a template	5’-GAGACGAGCTCCTCTTCTTGGATACCAGA-3’<br>5’-AGAGGAGCTCGTCTCTGGCTGTGATGGG-3’
